# Cancer-associated polybromo-1 bromodomain 4 missense variants variably impact bromodomain ligand binding and cell growth suppression

**DOI:** 10.1016/j.jbc.2024.107146

**Published:** 2024-03-07

**Authors:** Karina L. Bursch, Christopher J. Goetz, Guanming Jiao, Raymundo Nuñez, Michael D. Olp, Alisha Dhiman, Mallika Khurana, Michael T. Zimmermann, Raul A. Urrutia, Emily C. Dykhuizen, Brian C. Smith

**Affiliations:** 1 Department of Biochemistry, Medical College of Wisconsin, Milwaukee, Wisconsin, USA; 2 Structural Genomics Unit, Linda T. and John A. Mellowes Center for Genomic Sciences and Precision Medicine, Medical College of Wisconsin, Milwaukee, Wisconsin, USA; 3 Department of Medicinal Chemistry and Molecular Pharmacology, Purdue University, West Lafayette, Indiana, USA; 4 Clinical and Translational Sciences Institute, Medical College of Wisconsin, Milwaukee, Wisconsin, USA; 5 Department of Surgery, Medical College of Wisconsin, Milwaukee, Wisconsin, USA; 6 Program in Chemical Biology, Medical College of Wisconsin, Milwaukee, Wisconsin, USA

**Keywords:** bromodomains, cancer biology, cancer mutations, chromatin remodeling, epigenetics, genomics, histone acetylation, protein stability, site-directed mutagenesis, structure, function

## Abstract

The polybromo, brahma-related gene 1–associated factors (PBAF) chromatin remodeling complex subunit polybromo-1 (PBRM1) contains six bromodomains that recognize and bind acetylated lysine residues on histone tails and other nuclear proteins. PBRM1 bromodomains thus provide a link between epigenetic posttranslational modifications and PBAF modulation of chromatin accessibility and transcription. As a putative tumor suppressor in several cancers, PBRM1 protein expression is often abrogated by truncations and deletions. However, ∼33% of PBRM1 mutations in cancer are missense and cluster within its bromodomains. Such mutations may generate full-length PBRM1 variant proteins with undetermined structural and functional characteristics. Here, we employed computational, biophysical, and cellular assays to interrogate the effects of PBRM1 bromodomain missense variants on bromodomain stability and function. Since mutations in the fourth bromodomain of PBRM1 (PBRM1-BD4) comprise nearly 20% of all cancer-associated PBRM1 missense mutations, we focused our analysis on PBRM1-BD4 missense protein variants. Selecting 16 potentially deleterious PBRM1-BD4 missense protein variants for further study based on high residue mutational frequency and/or conservation, we show that cancer-associated PBRM1-BD4 missense variants exhibit varied bromodomain stability and ability to bind acetylated histones. Our results demonstrate the effectiveness of identifying the unique impacts of individual PBRM1-BD4 missense variants on protein structure and function, based on affected residue location within the bromodomain. This knowledge provides a foundation for drawing correlations between specific cancer-associated PBRM1 missense variants and distinct alterations in PBRM1 function, informing future cancer personalized medicine approaches.

DNA wrapping around histone octamers facilitates the organization of eukaryotic nuclear chromatin into nucleosomes (1, 2). Chromatin structure and dynamics are primarily mediated by histone posttranslational modifications, which impact the transcriptional accessibility of the underlying DNA (3). Extensive study of histone modifications and the effector proteins that add (“writers”), bind (“readers”), and remove (“erasers”) these modifications have led to the “histone code” hypothesis, where the number, type, combination, location, and time-synchronized deposition of diverse histone modifications leads to contextual downstream transcriptional regulation (4). Histone lysine acetylation (Kac) is a particularly abundant modification (5) associated with euchromatin (6), thereby increasing DNA accessibility (6) and activating transcription (7, 8).

Histone Kac is recognized by evolutionarily conserved bromodomains, ∼110-aa protein “reader” modules (9). Structurally, bromodomains consist of a four-helix bundle (⍺_Z_-⍺_A_-⍺_B_-⍺_C_), where the ZA and BC loops connecting these helices form a hydrophobic Kac binding pocket (9, 10, 11). Within this binding pocket, a conserved Asn residue in the BC loop directly interacts with the acetyl oxygen atom of Kac through a hydrogen bond (9, 11). We have highlighted the centrality of this Asn for Kac recognition in the bromodomain-containing protein polybromo-1 (PBRM1) with Asn to Ala variants that disrupt PBRM1 bromodomain binding to endogenous acetylated protein targets (12). Humans encode 61 bromodomains across 46 bromodomain-containing proteins (13), and most bromodomains exist in tandem with another bromodomain or histone binding domain (9). Six tandem bromodomains are found in PBRM1, a nominative and chromatin-targeting component of the polybromo, brahma-related gene 1 (BRG1)-associated factors (PBAF) complex (14, 15, 16). As the PBAF complex is a member of the larger Switch/sucrose nonfermentable family of ATP-dependent chromatin remodeling complexes (14, 15, 16), PBRM1 bromodomains link epigenetic histone Kac to PBAF-mediated transcriptional regulation and alterations in chromatin accessibility (10, 15, 17, 18).

PBRM1 promotes genomic stability *via* DNA damage repair (19, 20), and we have observed a protective role of PBRM1 in oxidative stress (21). Notably, lysine-14 acetylation on histone H3 (H3K14ac) is a histone modification associated with DNA damage (22) and a primary binding target of PBRM1 bromodomains (12, 23, 24), suggesting that H3K14ac may directly recruit PBRM1 to assist with DNA damage repair. PBRM1 also regulates the expression of a subset of p53 target genes *via* binding acetylated K382 of p53 (25). Consistent with these protective nuclear functions, PBRM1 is a putative tumor suppressor in several cancer types (12, 24, 26), especially clear cell renal cell carcinoma (ccRCC), where PBRM1 is mutated in ∼40% of ccRCC cases (27, 28, 29). Consistent with a tumor-suppressive function (12, 24, 26), PBRM1-deficient ccRCC tumors upregulate hypoxia-inducible factor (30, 31) and angiogenesis pathway transcription (32, 33, 34) to enhance tumor vascularity (35, 36). Therefore, cancer-associated mutations can disrupt the regulatory roles held by PBRM1 in multiple aspects of cellular homeostasis.

Complementing the elevated tumor angiogenic gene signatures observed with PBRM1 mutations (32, 33, 34), PBRM1 mutations in ccRCC patients are associated with improved response to antiangiogenic agents (32, 37, 38), the current standard of care for metastatic ccRCC in combination with immune checkpoint blockade (ICB) (39, 40). Additionally, loss-of-function (LOF) PBRM1 mutations in ccRCC patients are associated with increased efficacy of ICB therapy (41, 42), where the WT protein is implicated in reduced response to ICB (41, 43, 44). However, other studies failed to corroborate the enhanced clinical response to ICB observed with PBRM1 LOF mutations (32, 33, 45). These discrepancies in clinical response may be explained by individual PBRM1 mutations not being functionally equivalent; instead, different classes of PBRM1 mutations may have unique impacts on overall protein stability and activity in the context of cancer.

ccRCC-related PBRM1 mutations most frequently lead to complete loss of protein expression (27, 46). However, PBRM1 missense mutations are present in ∼15% of ccRCC cases (12, 46), leading to the expression of full-length PBRM1 protein variants. We and others have found that PBRM1 missense mutations cluster within its six bromodomains and noted that select PBRM1 bromodomain missense variants exhibit reduced protein stability, Kac binding, and tumor suppressor function (12, 23). However, the biophysical attributes and cellular implications of cancer-associated PBRM1 bromodomain missense variants remain largely unexplored. As PBRM1 plays context-dependent roles in several aspects of cancer biology, elucidating the effects of cancer-associated PBRM1 bromodomain missense mutations on protein stability and biochemical activity is essential to further delineate the roles of PBRM1 in cancer and ICB therapeutic response.

Here, we used an array of computational, biophysical, and cellular assays to interrogate the effects of cancer-associated PBRM1 bromodomain missense variants on protein stability and function. Probing the genomic landscape of cancer-associated PBRM1 missense mutations, we found many missense mutations cluster within the fourth bromodomain (BD4) of PBRM1 (PBRM1-BD4). Combining patient-derived mutational data with bromodomain residue conservation, we identified 16 missense variants for further analysis. We found cancer-associated PBRM1-BD4 missense variants variably impact protein stability, Kac binding ability, and cell growth suppression in a manner dependent on the affected residue location within the bromodomain. Taken together, our data suggest that cancer-associated PBRM1-BD4 missense variants lead to the expression of full-length proteins with variable stability, biochemical activity, and cellular function. Moreover, our data indicate that further characterization of PBRM1 bromodomain missense variants in the context of cancer pathogenesis and therapeutic response is mechanistically and clinically warranted to improve precision medicine approaches for cancer treatment.

## Results

### Cancer-associated missense mutations are overrepresented in the BD4 of PBRM1 and cluster in key structural and functional regions

**Figure 1:**
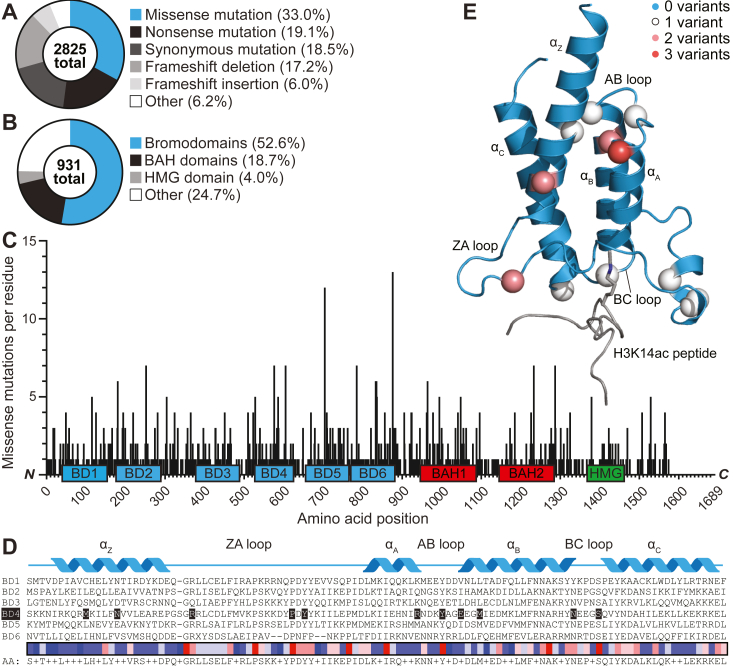
**PBRM1 incurs frequent missense mutations in the context of cancer.***A*, proportion of cancer-associated PBRM1 variants by mutation type, as annotated in the COSMIC (Catalog of Somatic Mutations in Cancer) database. *B*, percentage of cancer-associated missense mutations of PBRM1 by functional domain, as annotated in the COSMIC database. *C*, cancer-associated missense mutations of PBRM1-BD4 per residue across the entire peptide sequence, as annotated in the COSMIC database. Bromodomains (BD; *blue*), bromo-adjacent homology (BAH; *red*), and high-mobility group (HMG; *green*) domains are denoted, with domain boundaries determined from Pfam annotations ± 15 aa residues. *D*, structure-based sequence alignment of PBRM1 bromodomains, with the position of the four bromodomain ⍺-helices (*blue*) shown above. BD4 and the residues studied herein are highlighted within the sequence alignment. The heat map demonstrates the conservation level per residue across the six PBRM1 bromodomains, where higher conservation is indicated by *reds* and lower conservation is indicated by *blues*. *E*, Rosetta flexible peptide docking of an H3K14ac peptide (*gray*) from PBRM1-BD2 (PDB ID 2KTB) (79) to PBRM1-BD4 (PDB ID 3TLP) (9); mutated residues are represented as *spheres* and color-coded by the number of unique missense variants per residue examined in this study. H3K14ac, lysine-14 acetylation on histone H3; PBRM1, polybromo-1.

To define the cancer-associated mutational landscape affecting the PBRM1 gene, we curated the incidence of all cancer-associated PBRM1 missense mutations identified by next-generation sequencing (47). Missense mutations are the most abundant class (931 total, 33%) of all genetic alterations to PBRM1 (Fig. 1*A*). Moreover, missense mutations are concentrated (53%) in bromodomains (Fig. 1*B*), the histone Kac binding modules that comprise 41% of the protein sequence and are essential for overall protein activity (12, 23, 24). Furthermore, by mapping cancer missense mutations across the protein sequence, which largely follows domain architecture (Fig. 1*C*), we found that, despite comprising only 6% of the overall protein sequence, the BD4 harbors 10% of all cancer-associated PBRM1 missense mutations. PBRM1-BD4 is particularly important for recognizing endogenous PBRM1-acetylated protein targets [*e.g.*, histone H3 K14 (H3K14ac), p53 K382 (p53K382ac)] and subsequent PBRM1 biological activity (23, 24, 25). Therefore, we next determined the biophysical and cellular impacts of cancer-associated PBRM1-BD4 missense mutations.

For this purpose, we initially performed a structure-based sequence alignment of PBRM1-BD4 against the other five PBRM1 bromodomains to generate a bromodomain consensus sequence and residue conservation scores (Fig. 1*D*). Pairing this data with the known cancer-associated missense mutation frequency per residue (Fig. 1*C*), we selected 16 (17%) of the identified cancer-associated PBRM1-BD4 missense variants across 11 unique amino acid residues for recombinant expression and purification from *Escherichia coli* as isolated BD4 constructs, followed by *in vitro* biophysical characterization based on the criteria of observed in ≥3 patients, location at a conserved residue, or both (Table S1). Six of these variants are in the ZA or BC loops that form the histone Kac binding pocket (9, 10, 11) (Fig. 1*E*). The mutated residues of the remaining ten variants are in the α_Z_, α_A_, and α_B_ helices or the AB loop, regions that contribute to bromodomain core helical bundle folding and overall stability (Fig. 1*E*) (9). Because our variant selection criteria focused on residues both proximal and distal to key structural and functional regions of PBRM1-BD4 (Fig. 1*E*), we hypothesized that the 16 PBRM1-BD4 missense variants would variably impact stability and Kac binding capacity relative to PBRM1-BD4 WT.

### Most cancer-associated PBRM1-BD4 missense variants exhibit decreased protein stability but maintain overall secondary and tertiary structure

**Figure 2:**
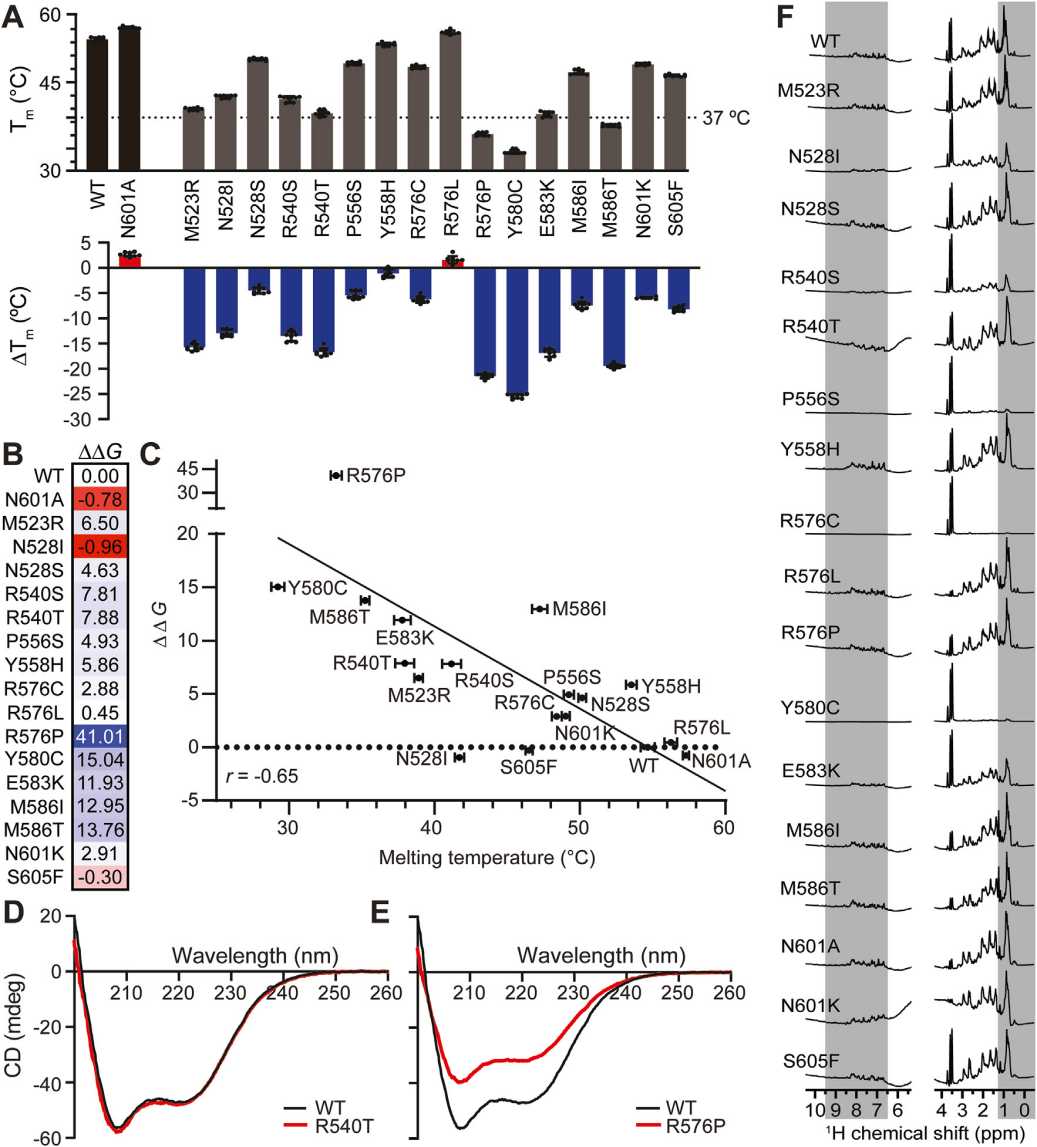
**Cancer-associated PBRM1-BD4 missense variants primarily exhibit decreased protein stability with intact secondary and tertiary structure integrity.***A*, PBRM1-BD4 missense variant *T*_m_ determined by SYPRO Orange thermal shift assay (controls shown in *light gray*, cancer-associated PBRM1-BD4 missense variants in *dark gray*); *ΔT*_m_ of PBRM1-BD4 variants compared to WT is also demonstrated (negative *ΔT*_m_ denoted in *blue*, positive Δ*T*_m_ in *red*), where error bars represent SD; n = 9 for PBRM1-BD4 WT and all missense variants except N601K, where n = 6. *B*, heat map indicates a change in Gibbs free energy (ΔΔ*G*) of PBRM1-BD4 missense variants compared to WT estimated by Rosetta modeling software (more divergent values shown in *blue*, less divergent in *red*). *C*, correlation of SYPRO Orange thermal shift assay and ΔΔ*G* datasets, where horizontal error bars represent SD of protein melting temperatures determined by the SYPRO Orange thermal shift assay. *D*, CD spectrum of PBRM1-BD4 missense variant R540T. *E*, CD spectrum of PBRM1-BD4 missense variant R576P. *F*, ^1^H-NMR spectra of PBRM1-BD4 WT and cancer-associated PBRM1-BD4 missense variants. The *gray highlighted regions* correspond to the spectral regions (backbone amide proton ∼6.5–9.5 ppm; saturated alkane methyl proton ∼0–1.25 ppm) used to assess variant tertiary structural integrity (56, 57). BD4, fourth bromodomain; CD, circular dichroism; ^1^H-NMR, one-dimensional proton NMR spectroscopy; PBRM1, polybromo-1; *T*_m_, protein melting temperature.

We expressed and purified the 16 selected cancer-associated PBRM1-BD4 missense variants as individual BD4 constructs for *in vitro* analysis of their structural stability and folding integrity. As protein melting temperature (*T*_m_) is a measure of protein stability (48), we used the SYPRO Orange thermal shift assay to determine the *T*_m_ and stability of the 16 missense variants compared to PBRM1-BD4 WT. Overall, cancer-associated PBRM1-BD4 missense variants are destabilized (average of variants *T*_m_ = 44.3 ± 8.2 °C; Δ*T*_m_ = −10.4 °C) relative to WT (*T*_m_ = 54.7 ± 0.5 °C) (Fig. 2*A*, Table S2). The Y580C variant displayed the greatest structural destabilization (*T*_m_ = 29.2 ± 0.4 °C; Δ*T*_m_ = −25.4 °C) consistent with its disruption of a conserved residue in the PBRM1-BD4 AB loop required for loop-helix fold stability (9) (Fig. 1*D*). Conversely, a control variant (N601A) at the conserved BC loop Asn residue directly involved in histone Kac binding (9, 11), exhibited slight stabilization (*T*_m_ = 57.3 ± 0.2 °C; Δ*T*_m_ = +2.6 °C). Only one cancer-associated variant (R576L) exhibited structural stabilization (*T*_m_ = 56.2 ± 0.4 °C; Δ*T*_m_ = +1.6 °C) (Fig. 2*A*, Table S2). Notably, six variants had *T*_m_ values below (R576P, Y580C, and M586T) or within 2 °C (M523R, R540T, and E583K) of 37 °C, the human body temperature (Fig. 2*A*, Table S2), indicating variant destabilization sufficient for partial unfolding under physiological conditions.

We also applied computational and experimental biophysical approaches to corroborate our SYPRO Orange thermal shift assay data. We measured cancer-associated PBRM1-BD4 missense variant *T*_m_ values with nano differential scanning fluorimetry to ensure the SYPRO Orange dye did not affect protein stability (Table S3). PBRM1-BD4 variant *T*_m_ derived by nano differential scanning fluorimetry correlated well with *T*_m_ determined from SYPRO Orange thermal shift assays (*r* = 0.99) (Fig. S1). Computational modeling of change in Gibbs free energy (ΔΔ*G*) is also an established predictor of how missense variants affect protein stability, where energetic differences between folded and unfolded protein states (Δ*G*_folding_) allow for the estimation of free energy changes between missense variant and WT proteins (49, 50, 51). As the sheer abundance of genetic missense variant data available makes it difficult to experimentally determine the functional impacts of every disease-implicated genetic missense variant, computational predictions of missense variant impacts on protein stability *via* ΔΔ*G* calculations and other methods can provide accelerated insight into potential mechanisms of pathogenicity for cancer-associated missense variants (52), particularly in patient diagnosis and treatment. Accordingly, we used molecular mechanic calculations to estimate the ΔΔ*G* of cancer-associated PBRM1-BD4 missense variants compared to the WT (Fig. 2*B*). This analysis demonstrated a negative correlation (*r* = −0.65) between thermal shift and ΔΔ*G* datasets (Fig. 2*C*), indicating that both methods are reliable measures of protein stability. Thus, the results of our complementary *in vitro* and *in silico* stability assays suggest that cancer-associated PBRM1-BD4 missense variants with affected residues in either the Kac binding loops or the structural core are sufficient to destabilize PBRM1-BD4 due to disruption of crucial bromodomain functional regions.

To assess the effects of the 16 PBRM1-BD4 missense variants on secondary structure integrity, we employed circular dichroism (CD). A largely α-helical protein exhibits characteristic CD spectral minima at 208 and 222 nm, based on the differential absorption of circularly polarized light by α-helices compared to other secondary structure elements (53). Our CD spectra demonstrate that the characteristic α-helical bromodomain secondary structure was retained in nearly all variants relative to the WT, with the R540T variant representative of these results (Figs. 2*D* and S2). However, a decrease in α-helical character was observed for the R576P variant (Fig. 2*E*), consistent with introducing a “helix-breaking” Pro residue (54, 55) into the PBRM1-BD4 α_A_ helix. Given the observed secondary structure retention by CD in the setting of thermal destabilization, cancer-associated PBRM1-BD4 missense variants effects on bromodomain stability and folding are more local than global.

To probe the effects of the 16 PBRM1-BD4 missense variants on PBRM1-BD4 tertiary structure integrity, we used one-dimensional proton NMR spectroscopy (^1^H-NMR). In ^1^H-NMR experiments, distinct and well-dispersed signals in the backbone amide proton (∼6.5–9.5 ppm) and saturated alkane methyl proton (∼0–1.25 ppm) regions are indicative of a well-folded protein (56, 57). Only missense variants R540S, P556S, R576C, Y580C, and E583K exhibited a loss of ^1^H-NMR signal in these key spectral regions, consistent with spectral broadening upon protein tertiary structure unfolding and/or protein aggregation (Fig. 2*F*). However, the majority of the PBRM1-BD4 missense variants retained intact tertiary structure (Fig. 2*F*), corroborating the intact PBRM1-BD4 missense variant secondary structure observed by CD.

### Cancer-associated PBRM1-BD4 missense variants decrease bromodomain acetyl-lysine binding

**Figure 3:**
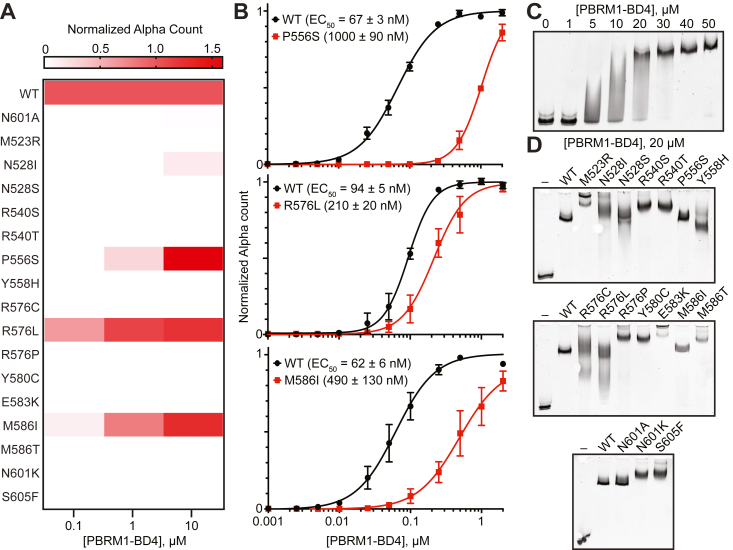
**Cancer-associated PBRM1-BD4 missense variants exhibit variable ligand binding capacity.***A*, heat map indicates PBRM1-BD4 variant (0.1–10 μM) binding of H3K14ac peptide (50 nM) compared to WT in AlphaScreen assays (n = 3). *B*, AlphaScreen titrations of PBRM1-BD4 P556S, M586I, and R576L (0.001–2 μM) against a biotinylated histone H3K14ac (50 nM) peptide (n = 3), where error bars represent SEM. *C*, EMSA of PBRM1-BD4 WT (0–50 μM) binding to 150 nM Widom 601 DNA (representative of n = 2). *D*, EMSA of PBRM1-BD4 variants (20 μM) binding to 150 nM Widom 601 DNA (representative of n = 2). Alpha, amplified luminescent proximity homogeneous assay; BD4, fourth bromodomain; EMSA, electrophoretic mobility shift assay; H3K14ac, lysine-14 acetylation on histone H3; PBRM1, polybromo-1.

Apart from altered protein stability and/or folding, cancer-associated missense variants can also exhibit differential functional activity (*i.e.*, gain-of-function or LOF mutations). As PBRM1 bromodomains bind H3K14ac (12, 23, 24), a histone posttranslational modification associated with active transcription (23, 24) and DNA damage (22), we developed a sensitive AlphaScreen (Alpha = amplified luminescent proximity homogeneous assay) binding assay (58, 59) to directly assess Kac binding capacity (EC_50_). We screened PBRM1-BD4 missense variants (0.1–10 μM) for their ability to bind an H3K14ac peptide (50 nM) relative to the WT (Figs. 3*A* and S3, Table S4). We also used a control N601A variant, which exhibits complete loss of H3K14ac peptide binding activity (Figs. 3*A* and S3, Table S4), consistent with the requirement of the amide nitrogen from the conserved Asn sidechain to form a hydrogen bond with the acetyl-lysine carbonyl oxygen (9, 11). We also observed ablation of Kac peptide binding for ∼75% of the missense variants (Fig. 3*A*).

Interestingly, the Y558H variant displayed minimal protein destabilization (Δ*T*_m_ = −1.1 °C) (Fig. 2*A*), whereas it lost Kac peptide binding (Fig. 3*A*). In contrast, the N528I variant incurred significant protein destabilization (Δ*T*_m_ = −12.9 °C) (Fig. 2*A*), but retained detectable Kac peptide binding at 10 μM (Fig. 3*A*). To determine the relative binding affinity of variants that maintained the ability to recognize H3K14ac peptide (defined as ≤1 μM PBRM1-BD4 protein with 50 nM H3K14ac peptide showing AlphaScreen counts above background), we performed full titrations of select PBRM1-BD4 missense variants (0.001–2 μM) and compared them to WT (Fig. 3*B*). While variants P556S (1000 ± 90 nM), R576L (210 ± 20 nM), and M586I (490 ± 130 nM) retained H3K14ac peptide binding, affinity was reduced ∼2- to 15-fold (Fig. 3*B*). The results of stability studies and Kac binding experiments suggest that cancer-associated PBRM1-BD4 missense variants impacting residues in either the Kac binding loops or the structural core are sufficient to decrease PBRM1-BD4 Kac by disrupting key bromodomain functional regions.

### PBRM1-BD4 and cancer-associated missense variants exhibit nucleic acid binding

We and others recently showed that PBRM1 bromodomains bind nucleic acids at a site overlapping the canonical Kac binding site (60). Several residues defining the putative PBRM1-BD4 nucleic acid binding pocket (60) are near (*e.g.*, R522, Y555, H599) or include (E583) the residues we consider in the cancer-associated PBRM1-BD4 missense variants analyzed in this study (*i.e.*, M523R, P556S, E583K, and N601K). Therefore, we employed electrophoretic mobility shift assays (EMSAs) to assess the impact of the 16 cancer-associated PBRM1-BD4 missense variants on binding to Widom 601 DNA, a classical nucleosome positioning DNA sequence used to probe *in vitro* chromatin dynamics (61). After titrating 150 nM Widom 601 DNA with PBRM1-BD4 WT to determine the optimal PBRM1-BD4 protein concentration required to approximate the nucleic acid EC_50_ (Fig. 3*C*), PBRM1-BD4 missense variants were screened for their ability to bind Widom 601 DNA (150 nM) relative to the WT protein (Figs. 3*D* and S4). All PBRM1-BD4 missense variants exhibited a nucleic acid binding capacity greater than or equal to that of the WT (Figs. 3*D* and S4). Thus, PBRM1-BD4 missense variants may contribute to cancer pathogenicity by increasing nonspecific PBRM1 chromatin binding capacity.

### Structural analysis provides rationales for PBRM1-BD4 missense variant impacts

**Figure 4:**
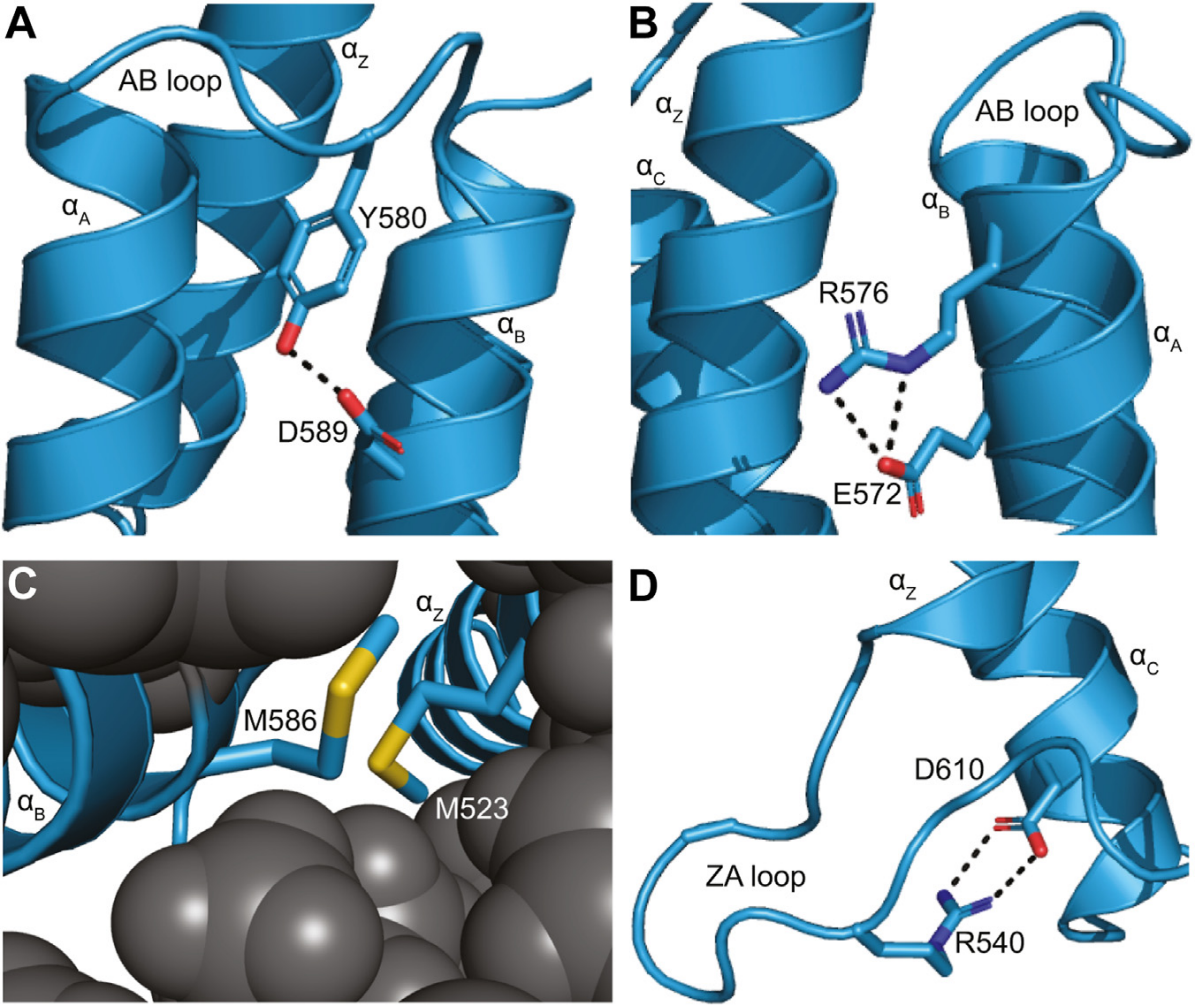
**Structural insights into the functional effects of cancer-associated PBRM1-BD4 missense variants.***A*, conserved residue Y580 stabilizes the loop-helix fold between the AB loop and the adjacent α_B_ helix. *B*, frequently mutated residue R576 helps maintain the structural integrity of the α_Z_ helix. *C*, frequently mutated residues M523 and M586 in the α_Z_ and α_B_ helices contribute to the stability of the PBRM1-BD4 α-helical core. *D*, conserved residue R540 contributes to the histone Kac binding pocket and adjacent α_C_ helix stability. BD4, fourth bromodomain; PBRM1, polybromo-1.

We next evaluated the structural and energetic features of PBRM1-BD4 WT protein relative to cancer-associated PBRM1-BD4 missense variants. These parameters help us to understand the impacts of specific PBRM1-BD4 missense variants on protein stability and ligand binding. Results from biophysical computations predicted that mutation at residues M523, R576, Y580, and M586 yield cancer-associated PBRM1-BD4 missense variants with the greatest instability (Fig. 2*B*), corroborating our *in vitro* protein stability results (Fig. 2*A*). Consistent with the conserved role of Y580 in stabilizing the loop-helix fold between the AB loop and the adjacent α_B_ helix (9) *via* hydrogen bonds with D589 (Fig. 4*A*), the Y580C variant is destabilized relative to the WT by nearly 25 °C (Fig. 2*C*). Additionally, analysis of the contributing energy terms to the ΔΔ*G* calculations demonstrates that the Y580C variant adversely increases the free energy of hydrogen bonding and Lennard-Jones attractive interactions (Fig. S5).

Within the α_A_ helix, the R576P variant is destabilized compared to other mutations at this site (Fig. 2*C*) due to substantial α-helical secondary structure disruption (Figs. 2*E* and 4*B*) that unfavorably increases Lennard-Jones repulsive interactions, the internal energy of sidechain rotamers, and proline ring closure energy (Fig. S5). At the same site, both the R576C and R576L variants exhibited minimal structural destabilization, likely because Cys and Leu possess greater helical propensity than Pro (54, 55) and fewer adverse impacts on folding energies (Fig. S5).

Consistent with the requirement for a hydrophobic residue at position 586 in the α_B_ helix to facilitate α-helix-helix packing (9), the M586T variant exhibits structural destabilization by an unfavorable increase in Lennard-Jones attractive interactions in the core bromodomain helical bundle (Figs. 4*C* and S5). Similarly, mutation of M523 in the α_Z_ helix to Arg impairs helix-helix packing, which increases helix solvation energy and rotamer internal energy at the Arg sidechain (Figs. 4*C* and S5). However, maintenance of the required hydrophobic residue at position 586 in the M586I variant (Fig. 4*C*) likely accounts for the decreased structural destabilization of M586I relative to M586T (Fig. 2*A*). This leads to the maintenance of histone Kac binding capacity by the M586I missense variant (Fig. 3, *A* and *B*).

We also performed EMSA analyses to determine PBRM1-BD4 missense variant DNA binding. Notably, we find that although the PBRM1-BD4 E583K substitution lies within the putative PBRM1-BD4 nucleic acid binding pocket (60), it maintains nucleic acid binding relative to WT (Figs. 3*D* and S4). This phenomenon is likely due to the additional positive charge introduced by the lysine residue in the PBRM1-BD4 E583K variant, which enhances variant-nucleic acid electrostatic interactions (62). Notably, PBRM1 bromodomains possess an increased binding affinity for histone Kac when bound to RNA (60), suggesting that cancer-associated PBRM1 bromodomain missense variants may exhibit altered functions not only in terms of nucleic acid binding in the context of chromatin but also transcriptional regulation.

We also demonstrate that mutations at R540 impact both bromodomain stability and Kac binding (Figs. 2*A* and 3*A*). In fact, R540 interacts through a salt bridge with D610 in the adjacent α_C_ helix in the histone Kac binding pocket (Fig. 4*D*). Additionally, missense mutations at R540 adversely increase the free energy of hydrogen bonding and coulombic electrostatic potential (Fig. S5). These results indicate that structural destabilization in the ZA and BC loops composing the PBRM1-BD4 histone Kac binding pocket may be sufficient to decrease *in vitro* histone Kac binding.

### Cancer-associated PBRM1-BD4 missense variants decrease acetyl-lysine binding in renal cancer cells

**Figure 5:**
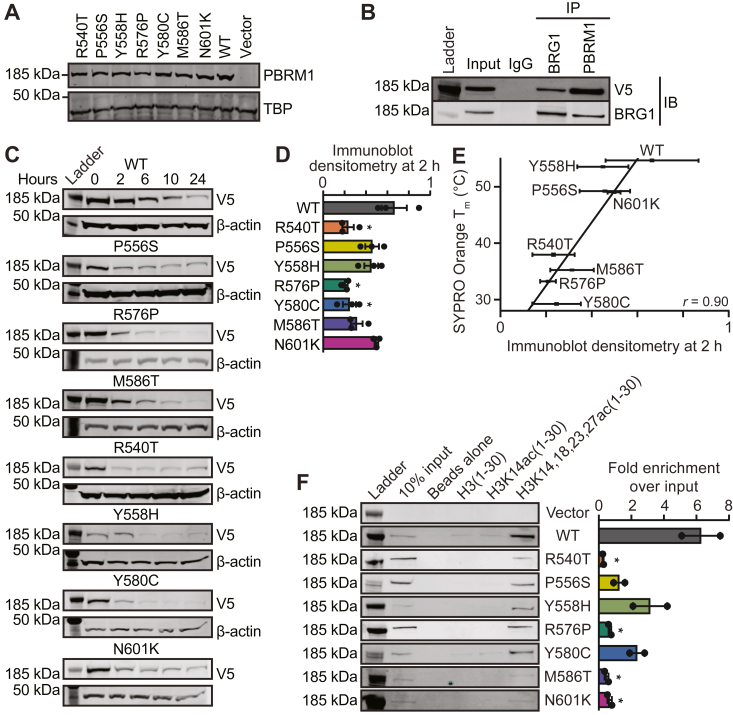
**Cancer-associated PBRM1-BD4 missense variants exhibit impaired stability and acetylated histone binding.***A*, immunoblot demonstrates equivalent expression of full-length PBRM1-BD4 missense variants in a Caki-2 tetracycline-inducible system. *B*, coimmunoprecipitation with BRG1 and V5-tagged PBRM1. *C*, immunoblots of V5-tagged PBRM1-BD4 WT and PBRM1-BD4 missense variants and beta-actin from Caki-2 cells treated with 100 μg/ml cycloheximide for 0, 2, 6, 10, or 24 h. *D*, immunoblot densitometry quantitation of PBRM1-BD4 WT and PBRM1-BD4 missense variants at 2 h of cycloheximide treatment. Significance was calculated using an unpaired Student’s *t* test, where ∗*p* < 0.05, and error bars represent the SEM. *E*, correlation of PBRM1-BD4 WT and PBRM1-BD4 missense variant protein stability as assessed by the cellular cycloheximide chase assay at 2 h and the biophysical SYPRO Orange thermal shift assay, where *horizontal error bars* represent SD of immunoblot densitometry quantitation at 2 h of cycloheximide treatment and *vertical error bars* represent SD of protein *T*_m_ values determined by the SYPRO Orange thermal shift assay. *F*, acetylated histone H3 peptide pulldown (n = 2) by PBRM1 WT and PBRM1-BD4 missense variants as measured by fold enrichment of H3K14,18,23,27ac(1–30) over input. Significance was calculated using an unpaired Student’s *t* test where ∗*p* < 0.05 and error bars represent SEM. BD4, fourth bromodomain; BRG1, brahma-related gene 1; PBRM1, polybromo-1; *T*_m_, protein melting temperature.

To validate the biophysical impacts of cancer-associated PBRM1-BD4 missense variants within the context of the full-length protein and cellular conditions, we used a lentiviral transduction system (63). We expressed full-length PBRM1 WT and seven full-length cancer-associated PBRM1-BD4 missense variants in Caki-2 ccRCC cells lacking endogenous PBRM1 (63). We selected seven cancer-associated PBRM1-BD4 missense variants based on their ability to disrupt PBRM1-BD4 stability, Kac binding, or both. Using immunoblotting, we confirmed that these eight Caki-2 cell lines express equivalent PBRM1 protein levels after doxycycline treatment (Fig. 5*A*). We also employed coimmunoprecipitation (64) with BRG1, the ATPase of the PBAF chromatin remodeling complex (16), to validate that our V5-tagged full-length PBRM1 construct effectively incorporated in the PBAF complex (Fig. 5*B*).

To complement our *in vitro* biophysical analysis of PBRM1-BD4 missense variant stability, we conducted a cycloheximide chase assay to evaluate time-dependent protein degradation in the setting of translational inhibition (65) as a proxy for PBRM1-BD4 missense variant stability in Caki-2 cells (Figs. 5*C* and S6*A*). PBRM1 WT protein levels persisted in Caki-2 cells with increasing cycloheximide exposure (Fig. 5*C*). While PBRM1-BD4 missense variants P556S, Y558H, and N601K initially maintained similar protein levels relative to PBRM1 WT at 2 h of cycloheximide exposure (Fig. 5, *C* and *D*), the protein levels of all seven PBRM1-BD4 missense variants decreased relative to PBRM1 WT with increasing cycloheximide exposure (Figs. 5*C* and S6, *A* and *B*). All seven PBRM1-BD4 missense variants possess diminished stability compared to PBRM1 WT in Caki-2 cells. Interestingly, we observed a surprisingly strong correlation between the results of the cellular cycloheximide chase assay and the biophysical SYPRO Orange thermal shift assay (Figs. 5*E* and S6*C*). This indicates that the decreased *in vitro* thermostability of recombinant PBRM1-BD4 missense variants directly affects the stability of the full-length PBRM1 protein within the nuclear PBAF chromatin remodeling complex. The *T*_m_ of an isolated PBRM1 bromodomain may therefore be a sufficient proxy for the overall stability of full-length PBRM1 protein in cells, providing key mechanistic information for patient diagnosis and treatment in the context of cancer.

We next tested the effects of cancer-associated PBRM1-BD4 missense variants on full-length PBRM1 histone Kac binding by incubating transduced Caki-2 nuclear lysates with biotin-labeled H3K14ac and H3K14/18/23/27ac peptides bound to streptavidin resin. The binding of the seven PBRM1-BD4 missense variants to H3K14/18/23/27ac peptides was decreased by 59 to 94% compared to the WT (Figs. 5*F* and S6*D*). Considering our previous data showing that BD4 is required for maximal PBRM1 protein affinity to Kac histone peptides (63), we conclude that missense variants impacting residues in the Kac binding regions or structural core of PBRM1-BD4 are sufficient to decrease full-length PBRM1 protein affinity for histone Kac ligands.

### Cancer-associated PBRM1-BD4 missense variants are defective renal cancer cell growth suppressors and decrease PBRM1-regulated gene expression

**Figure 6:**
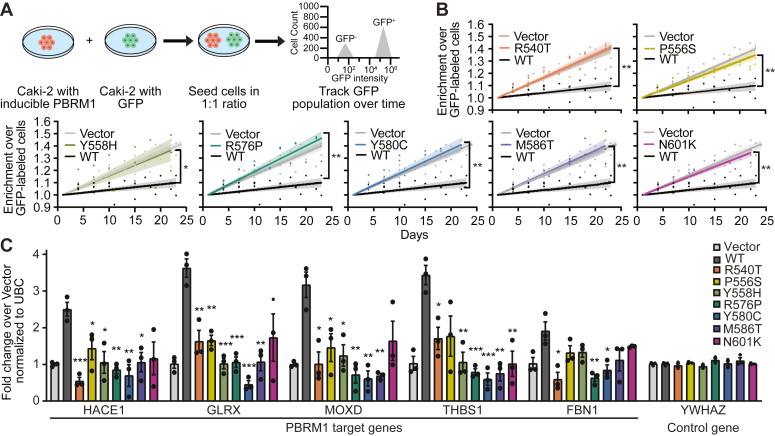
**Cancer-associated PBRM1-BD4 missense variants exhibit impaired cancer cell growth suppression and PBRM1 target gene regulation.***A*, workflow of the FACS cell proliferation competition assay. *B*, Caki-2 PBRM1 WT and PBRM1-BD4 missense variant cell growth over 22 days. Significant differences between cell lines were calculated using a two-way ANOVA (mixed model) with Tukey post hoc analysis where ∗*p* < 0.05 and ∗∗*p* < 0.01, and *colored wedges* around trendlines represent SD (n = 2 for vector, n = 4 for WT, n = 3 for missense variants). *C*, RT-qPCR (n = 3) of select PBRM1 target and nontarget genes. Significance was calculated using an unpaired Student’s *t* test where ∗*p* < 0.05, ∗∗*p* < 0.01, and ∗∗∗*p* < 0.001 and error bars represent the SEM. BD4, fourth bromodomain; FACS, fluorescence-activated cell sorting; PBRM1, polybromo-1; RT-qPCR, quantitative real-time PCR.

Consistent with the negative regulation of cell growth by PBRM1 observed in several systems (21, 23), we previously found that reexpression of PBRM1 WT in Caki-2 cells *via* lentiviral transduction reduces cell proliferation compared to vector-transduced cells (12, 60, 63). With this knowledge, we compared the growth of our seven GFP^-^ PBRM1-variant expressing and one GFP^-^ PBRM1 WT expressing Caki-2 cell lines to GFP^+^ Caki-2 cells using a fluorescence-activated cell sorting–based cell proliferation competition assay (Fig. 6*A*) (60). As with our previous observations (60), only PBRM1 WT-transduced Caki-2 cells did not outcompete the growth of GFP^+^ Caki-2 cells (Figs. 6*B*, S7). In contrast, all cancer-associated PBRM1-BD4 missense variant Caki-2 cells outcompeted the growth of GFP^+^ Caki-2 cells, similar to vector-transduced Caki-2 cells (Figs. 6*B* and S7). The results of these competition assays indicate that the selected PBRM1-BD4 missense variants decrease the ability of the whole protein to suppress Caki-2 ccRCC cell growth relative to the WT protein.

In the context of the ATP-dependent PBAF chromatin remodeling complex (14, 15, 16), the PBRM1 bromodomains provide a mechanistic link between epigenetic histone Kac and PBAF-mediated transcriptional regulation (10, 15, 17, 18). Therefore, we investigated the effects of cancer-associated PBRM1-BD4 missense variants on PBRM1-mediated regulation of *HACE1*, *GLRX*, *MOXD1*, *THBS1*, and *FBN1*. These five genes constitute part of our previously described gene signature regulated by PBRM1 (63). Functionally, these genes are implicated in tumor suppressor relevant-cellular signaling pathways ranging from cell adhesion to redox regulation (63). The results of these experiments demonstrate that all cancer-associated PBRM1-BD4 missense variants impair the ability of full-length PBRM1 to increase the expression of its target genes and do not affect the expression of non-PBRM1 target genes like *YWHAZ* (Fig. 6*C*). These data suggest that the deleterious effects of cancer-associated PBRM1-BD4 missense variants on whole-protein tumor suppressive function may be mediated by disturbances of PBRM1-mediated transcriptional regulation.

## Discussion

In this study, we employed an array of computational, biophysical, and cellular assays to comprehensively analyze the effects of PBRM1-BD4 missense variants on protein stability, structure, ligand binding, and cellular activity. This research contributes to our understanding of the biochemical mechanisms underlying the function of PBRM1 and its bromodomains within the PBAF chromatin remodeling complex. Indeed, we reveal that missense mutations are the most prevalent genetic alteration in the *PBRM1* gene, concentrated in the bromodomains and the BD4 in particular (Fig. 1, *A*–*C*). As PBRM1-BD4 is targeted by missense mutations in cancer, our findings shed light on the importance of BD4 in the mutational landscape of PBRM1 and its involvement in histone Kac interactions and cellular function. In addition, our *in silico* modeling and biophysical experiments demonstrate that cancer-associated PBRM1-BD4 missense variants typically result in decreased protein stability compared to the WT counterpart (Fig. 2, *A*–*C*). We describe variants displaying significantly reduced *T*_m_, indicating destabilization and partial unfolding of PBRM1-BD4 under physiological conditions. The Y580C variant shows the greatest structural destabilization among the variants, while the R576L variant showed slight stabilization (Fig. 2*A*). As Y580 is an important residue for PBRM-BD4 structure and function (9, 11), these findings underscore the variable impact of PBRM1-BD4 missense variants on protein stability.

We also investigated the effect of PBRM1-BD4 missense variants on Kac binding and found that they display an overall decreased but wide-ranging capacity to bind H3K14ac, a histone posttranslational modification associated with DNA damage (22) and transcriptional activation (23, 24). AlphaScreen binding assays reveal that some variants lost binding capacity for histone H3K14ac peptides, while other variants exhibited moderately reduced affinity compared to the WT (Figs. 3, *A* and *B* and S3). These results underscore that cancer-associated PBRM1-BD4 missense variants variably disrupt the ability of PBRM1 to recognize and bind its targets, thereby affecting its functional roles in cellular processes. We validated these findings at the cellular level, employing lentiviral transduction to express full-length PBRM1 WT and select PBRM1-BD4 missense variants in renal cancer cells (Fig. 5*A*). Our analysis demonstrated that cancer-associated PBRM1-BD4 missense variants variably impaired the cellular stability and histone Kac binding of full-length PBRM1 (Fig. 5, *C* and *F*) and diminished PBRM1-mediated cell growth suppression (Fig. 6*B*). This phenomenon is reflected by the fact that renal cancer cells expressing full-length PBRM1-BD4 variants did not suppress cell growth in a manner similar to renal cancer cells expressing PBRM1 WT (Fig. 6*B*). Moreover, our results confirm and expand upon the disruptive effects of cancer-associated variants at conserved residues, such as Y580 and N601, on PBRM1-BD4 histone Kac binding capacity (Fig. 3*A*) and whole-protein suppressive effects on ccRCC cell growth (Fig. 6*B*) that have been noted in previous studies (23).

In addition to protein stability and Kac binding, this study explored the impact of cancer-associated PBRM1-BD4 missense variants on nucleic acid binding and PBRM1-mediated transcriptional regulation. We observe that PBRM1-BD4 missense variants have comparable (or potentially increased) nucleic acid binding capacity compared to the WT protein (Figs. 3*D* and S4). However, PBRM1-BD4 missense variants impaired the ability of PBRM1 to regulate the expression of target genes relevant to tumor suppression (Fig. 6*C*). Since PBRM1 is a key chromatin-targeting subunit of the PBAF chromatin remodeling complex (14, 15, 16), the apparent increased nucleic acid binding of select cancer-associated PBRM1-BD4 missense variants may contribute to cancer pathophysiology through aberrant genomic targeting of the PBAF complex and altered transcriptional regulation (60, 66).

Our structural analyses (Fig. 4) provide further insights into the effects of distinct PBRM1-BD4 missense variants on protein stability and structure (Fig. 2), ligand binding (Fig. 3), and cellular activity (Figs. 5 and 6). These investigations reveal that mutations at structurally significant residues in PBRM1-BD4, such as M523, R576, Y580, and M586, cause local structural destabilization (Fig. 2, *A*–*C*) and disruption of histone Kac binding (Fig. 3*A*). We also define the role of specific residues, such as R540 and E583, in the histone Kac binding pocket and their impact on binding affinity and protein stability.

Although PBRM1 is often classified as a tumor suppressor (24, 25, 26), clinical observations correlating PBRM1 protein expression or mutational status with ICB and antiangiogenic response suggest that PBRM1 plays diverse roles in cancer (32, 33, 41, 42, 43, 44, 45, 67). Consequently, therapies targeting the PBRM1 bromodomains have garnered attention in drug discovery and experimental therapeutics (59, 68, 69, 70). Despite the loss of PBRM1 protein expression in many cases of cancer-associated PBRM1 mutations (46, 71), this study highlights the clinical relevance of cancer-associated PBRM1-BD4 missense variants with variable stability and histone Kac binding compared to the WT protein. The variants that retain some degree of WT function remain viable targets for novel bromodomain inhibitors, such as the selective and cell-active PBRM1 bromodomain inhibitors we recently developed (59). Thus, patients with functional PBRM1 protein variants may benefit clinically from treatment with selective PBRM1 bromodomain inhibitors in combination with standard-of-care ICB and antiangiogenic therapies. Therefore, understanding the effects of specific PBRM1 bromodomain missense variants on protein stability and function can inform precision medicine approaches targeting PBRM1 and its bromodomains in the context of cancer therapy.

Overall, the new knowledge provided by the current study advances the current understanding of the impact of cancer-associated PBRM1-BD4 missense variants on protein stability, Kac binding, and cell growth suppression. Moreover, our results uncover PBRM1-mediated molecular mechanisms disrupted by cancer-associated missense mutations and the potential for select missense variants retaining WT protein functions to serve as therapeutic targets in cancer. We are optimistic that the insights gained from these investigations will contribute to developing future precision medicine strategies that target PBRM1 and its bromodomain interactions for cancer treatment by facilitating the correlation of specific patient missense variants with distinct alterations in PBRM1 tumor suppressor functions and activities and clinical patient outcomes.

## Experimental procedures

### COSMIC database mining

Data comprising all known cancer-associated mutations in PBRM1 (ENST00000337303) identified by next-generation sequencing and curated in the Catalog of Somatic Mutations in Cancer (COSMIC) database were mined from COSMIC for analysis on July 11, 2023. The R540S variant was identified from earlier mining of COSMIC data (71). The number of mutations per mutation type was determined by recording the total number of entries listed in the COSMIC positive data table for each mutation type. The number of missense mutations per PBRM1 functional domain was determined by downloading the COSMIC positive data table of PBRM1 missense mutations and identifying which missense mutations fell within PBRM1 functional domains, with domain boundaries determined from Pfam annotations plus or minus an additional 15 aa residues beyond each domain boundary.

### Site-directed mutagenesis

The His_6_-tagged PBRM1-BD4 bromodomain construct (aa 496–637) in the pNIC-CTHF vector was a gift from Nicola Burgess-Brown (Addgene plasmid #39103). Cancer-associated missense mutations of PBRM1-BD4 were generated by site-directed mutagenesis (Biozilla) and confirmed by Sanger sequencing (72).

### Protein expression and purification

Recombinant PBRM1-BD4 WT, PBRM1-BD4 N601A, and cancer-associated PBRM1-BD4 missense variants were purified from BL21(DE3) *E. coli* by nickel-affinity chromatography. BL21(DE3) cells were transformed and grown at 37 °C in Luria-Bertani, 2×YT, or Terrific Broth media with 50 mg/L kanamycin to an *A* of ∼0.6 at 600 nm. Protein expression was induced overnight with 0.1 mM IPTG at 18 °C. Cells were harvested *via* centrifugation at 5000*g*, and cell pellets were frozen at −80 °C until lysis. Frozen cells were thawed on ice and resuspended in lysis buffer (50 mM Hepes, 500 mM NaCl, 5% v/v glycerol, 5 mM imidazole, pH 7.5). When reagents were available, 1:1000 aprotinin, E−64, leupeptin, bestatin, pepstatin A, and PMSF protease inhibitors were also added to the lysis buffer used to resuspend frozen cells. Resuspended cells were immediately lysed *via* sonication for 10 min (pulsed, amplitude 3.5, 50% work cycle), and lysates were clarified by centrifugation for 30 min at 30,000*g*. Clarified lysates were then applied to nickel-nitrilotriacetic acid (Ni-NTA) resin (0.75 ml resin/L culture) at 4 °C for at least 1 h while rocking. The protein-bound Ni-NTA resin was applied to a column, washed twice with 15 ml of lysis buffer, and eluted using increasing concentrations of imidazole in lysis buffer (5 ml of 50, 100, 150, 200, and 250 mM imidazole). Fractions were resolved by SDS-PAGE and those containing recombinant proteins of interest were pooled. Protein samples were further purified by gel filtration using an ENrich SEC 70 10 × 300 mm column (Bio-Rad, 7801070) into a storage buffer (50 mM Hepes, 500 mM NaCl, 5% v/v glycerol, pH 7.5).

For protein used in CD and EMSAs, clarified lysates were applied to Ni-charged MagBeads (GenScript, L00295) (∼1 ml beads/L culture) at 4 °C overnight while rocking. The protein-bound Ni-charged MagBeads were washed with 3 × 10 ml lysis buffer containing 0.02% v/v Tween 20, followed by 3 × 10 ml lysis buffer alone. Protein was eluted off the beads with 5 ml elution buffer (50 mM Hepes, 500 mM NaCl, 5% v/v glycerol, 150 mM imidazole, pH 7.5) using the AmMag SA Plus semiautomated purification system (GenScript, L01013). Protein samples were further purified by gel filtration using an Enrich SEC 70 10 × 300 mm column (Bio-Rad) or a Superdex 75 Increase 10/300 Gl column (Cytiva, 29148721) into storage buffer (50 mM Hepes, 500 mM NaCl, 5% v/v glycerol, pH 7.5) or EMSA buffer (52.4 mM K_3_PO_4_, 50 mM KCl, 5% v/v glycerol, 1 mM DTT, pH 7).

For protein used in NMR, BL21(DE3) cells were transformed and grown at 37 °C in Terrific Broth media with 50 mg/ml kanamycin, subcultured at 37 °C in 25 ml of medium P minimal media, and then grown at 37 °C in 500 ml of medium P minimal media with 50 mg/L kanamycin to an *A* of ∼0.6 at 600 nm. Protein expression was induced overnight with 0.1 mM IPTG at 18 °C. Cells were harvested and lysed as described above. Clarified lysates were applied to Ni-charged MagBeads (GenScript, L00295) (∼2 ml beads/L culture) at 4 °C overnight while rocking. The protein-bound Ni-charged MagBeads were washed with 3 × 10 ml lysis buffer containing 0.02% v/v Tween 20, followed by 3 × 10 ml lysis buffer alone. Protein was eluted off the beads with 5 ml elution buffer (50 mM Hepes, 500 mM NaCl, 5% v/v glycerol, 200 mM imidazole, pH 7.5) using the AmMag SA Plus semiautomated purification system (GenScript, L01013). Eluted protein was exchanged into tobacco etch virus (TEV) protease cleavage buffer (10 mM Tris–HCl, 150 mM NaCl, 0.5 mM EDTA, pH 8 at 20 °C) using PD-10 columns packed with Sephadex G-25 resin (Cytiva, 17085101). Desalted protein was then incubated with TEV protease overnight at 4 °C while rocking in 5 ml of TEV protease cleavage buffer supplemented with 1 mM DTT. TEV-cleaved protein was then applied to Ni-NTA resin (2 ml resin/L culture) at 4 °C for at least 2 h while rocking. The protein-bound Ni-NTA resin was applied to a column, washed five times with 5 ml of lysis buffer, and TEV protease was eluted with 5 ml and 10 ml of elution buffer. Fractions were resolved by SDS-PAGE and those containing recombinant cleaved proteins of interest were pooled. Protein samples were further purified by gel filtration using a Superdex 75 Increase 10/300 Gl column into PBRM1-BD4 NMR buffer (50 mM K_3_PO_4_, 50 mM KCl, 1 mM DTT, 0.2% w/v NaN_3_, pH 6.8).

For all protein purifications, monomeric protein was collected based on the chromatographs resulting from gel-filtration size-exclusion chromatography. Concentrations of purified proteins were determined by the method of Bradford using bovine serum albumin as a standard (73), aliquoted, flash-frozen, and stored at −80 °C.

### SYPRO Orange thermal shift

PBRM1-BD4 proteins (15 μM) were combined with 5 × SYPRO Orange dye (Sigma-Aldrich, S5692) in a 30 μl reaction volume. Reactions were immediately added to a 96-well PCR plate, and protein melting curves were monitored by SYPRO Orange fluorescence over a temperature gradient of 25 to 95 °C using an Mx3005P PCR instrument (Stratagene). Fluorescence values measured before the minimum and after the maximum were excluded from curve fitting, and the resulting curve was fit using the Boltzmann Sigmoidal (Equation 1) using GraphPad Prism (https://www.graphpad.com):

(Eq. 1) $$y=LL+\frac{UL-UL}{1+\exp\left(\frac{T_{m}-X}{a}\right)}$$

where UL and LL are the maximum and minimum fluorescence values, respectively; *a* is the slope of the curve within the melting range, and *T*_m_ is the melting temperature.

### Nano differential scanning fluorimetry

PBRM1-BD4 proteins were prepared at a concentration of 30 μM in buffer (50 mM Tris–HCl pH 7.5, 500 mM NaCl, 10% v/v glycerol, 1 mM tris(2-carboxyethyl)phosphine) for a final volume of 40 μl. Samples were drawn up in triplicate in Prometheus NT.48 high-sensitivity capillaries (NanoTemper Technologies, PR-C006) and run at 100% excitation power with a temperature ramp of from 20 °C to 95 °C increasing 1 °C/min in a Prometheus NT.48 instrument (PR001). As PBRM1-BD4 lacks Trp residues, intrinsic Tyr fluorescence at 330 nm was plotted *versus* temperature, and PBRM1-BD4 protein *T*_m_ was determined by identifying the maximum of the first derivative of the fluorescence signal with ThermControl software version 2.3.1 (NanoTemper Technologies, https://nanotempertech.com/prometheus/nt48-software).

### Circular dichroism

PBRM1-BD4 secondary structure was assessed using a J-1500 CD spectrophotometer (JASCO). The samples were prepared at a concentration of 0.4 mg/ml in buffer (50 mM Na_3_PO_4_, 200 mM NaF, 5% v/v glycerol, pH 7.5 at 20 °C) and placed in 1 mm quartz cuvettes (Thermo Fisher Scientific). CD spectra were recorded at 25 °C from 280 to 200 nm, with a data pitch of 0.1 nm. A bandwidth of 1 nm was used with a digital integration time of 1 s and a scanning speed of 50 nm/min. Each spectrum was accumulated from five scans and corrected by subtracting the buffer spectrum from the sample spectrum. The data was processed using Spectra Analysis software supplied by the manufacturer (JASCO, https://jascoinc.com/products/spectroscopy/molecular-spectroscopy-software/) and transferred to GraphPad Prism for presentation.

### NMR spectroscopy

PBRM1-BD4 proteins were prepared at a concentration of 13 to 111 μM in PBRM1-BD4 NMR buffer (50 mM K_3_PO_4_ pH 6.8, 50 mM KCl, 1 mM DTT, 0.2% w/v NaN_3_) and 4.4 to 5% v/v D_2_O. ^1^H-NMR data was collected at 25 °C on a Bruker Avance II 600 MHz spectrometer equipped with a triple resonance *z*-axis gradient cryoprobe and SampleJet autosampler, which allowed automatic tuning, matching, and shimming for each sample. ^1^H-NMR experiments consisted of 128 scans for PBRM1-BD4 WT and all missense variants except for variants P556S, R576C, and Y580C, where the scan number was increased to maintain an equivalent signal-to-noise ratio for proteins analyzed at lower concentrations. Spectra were processed with MNova from NMRBox (https://mestrelab.com/software/mnova-software/) (74).

### Rosetta protein modeling

X-ray structure coordinates for apo PBRM1-BD4 were obtained from the Protein Data Bank (PDB ID 3TLP) (9). All calculations were performed using the Rosetta 3.9 software release (https://www.rosettacommons.org/home). The ddg_monomer application (49) was used to predict changes in Gibbs free energy induced by point mutations using the high-resolution protocol, which uses the following flags: -ddg:weight_file soft_rep_design, -ddg::iterations 50, -ddg::local_opt_only false, -ddg::min_cst true, -ddg::sc_min_only false, and -ddg::ramp_repulsive true. Rosetta flexible peptide docking was performed using the FlexPepDock application refinement protocol (75), which uses the following flags: -nstruct 1000, -ex1, -ex2aro, and -flexPepDocking:pep_refine true. Input complexes were prepared by superimposing the structure of PBRM1-BD2 bound to each of the twenty solution NMR states of an acetylated histone H3 peptide (sequence ARTKQTARKSTGGK(acetyl)APRKQL, PDB ID 2KTB) onto PBRM1-BD4. All visualizations were prepared using PyMOL (Version 2.0.5, https://pymol.org/).

### AlphaScreen

All binding assays were conducted in light gray, half area 96-well plates (PerkinElmer, 6002350) in a total volume of 40 μl. All stock solutions were prepared in the assay buffer comprised of 1× AlphaLISA Epigenetics buffer (PerkinElmer, 5× AL008C/F) with 0.05% v/v Tween 20 and 2 μM tris(2-carboxyethyl)phosphine. For initial screens, each recombinant His_6_-tagged PBRM1-BD4 mutant was incubated at three concentrations (0.1 μM, 1 μM, and 10 μM) with 50 nM biotinylated histone H3K14ac(1–20) peptide (NH_2_-ARTKQTARKSTGGK(acetyl)APRKQLK(biotin)-CONH_2_; Peptide 2.0). Ten microliters of a 4 × (200 nM) biotinylated histone H3K14ac peptide stock solution and 10 μl of 4 × (40, 4, 0.4 μM or 0.004–8 μM) protein stock solutions were added to each well. The plate was then incubated for 30 min at room temperature. A bead solution comprising 8 μg/ml streptavidin donor beads and 8 μg/ml nickel-chelate acceptor beads (PerkinElmer, AlphaScreen Histidine (Nickel Chelate) Detection Kit, 500 assay points, 6760619C) was prepared in assay buffer. Twenty microliters of bead solution was added to each well under reduced light, and the plate was covered and incubated for an additional hour in the dark. Luminescence was subsequently read on a BioTek Cytation 5 imaging reader (Agilent, 16277) using the Alpha filter cube (Agilent, 1325000), and Alpha counts were analyzed using GraphPad Prism.

### Electrophoretic mobility shift assays

Five percent 75:1 acrylamide:bisacrylamide native gels were set and prerun in chilled 0.2 × Tris-borate-EDTA buffer on ice at 4 °C for 60 min at 125 V. Samples were prepared by mixing 150 nM Widom 601 DNA (a gift from Dr Emma Morrison, Department of Biochemistry, Medical College of Wisconsin (76)) with 0 to 50 μM PBRM1-BD4 WT or 20 μM PBRM1-BD4 missense variants purified into EMSA buffer (final assay concentration 10.5 mM K_3_PO_4_ pH 7 at 20 °C, 10 mM KCl, 1% v/v glycerol, 0.2 mM DTT). Samples were equilibrated on ice for 1 h. The samples were then mixed with an equivalent volume of 2 × loading dye (10% w/v sucrose and 0.02% v/v bromophenol blue in 0.5 × Tris-EDTA buffer). Samples were loaded and ran on the prerun native gels in chilled 0.2 × Tris-borate-EDTA buffer on ice at 4 °C for 45 to 60 min at 125 V. Gels were stained with ethidium bromide and visualized using a ChemiDoc MP imaging system (Bio-Rad).

### Cell culture conditions

All cells were purchased from American Type Culture Collection (ATCC) and only used if cultured for fewer than 30 passages. All cells were screened for *mycoplasma* (Lonza, MycoAlert) on a weekly basis. Caki-2 cells (ATCC) were cultured in McCoy’s 5A medium (Corning Mediatech, Inc) supplemented with 10% fetal bovine serum (Corning Mediatech, Inc), 1% Minimum Essential Medium nonessential amino acids (Corning Mediatech, Inc), 1% antibiotics (100 units/ml penicillin and 100 μg/ml streptomycin (Corning Mediatech, Inc), 2 mM L-alanyl-L-glutamine (Corning glutagro; Corning Mediatech, Inc), and 2.5 μg/ml plasmocin (InvivoGen, Inc). HEK-293T (ATCC) were cultured in DMEM (Corning Mediatech, Inc) supplemented with 10% fetal bovine serum (Corning Mediatech, Inc), 1% antibiotics (100 units/ml penicillin and 100 μg/ml streptomycin; Corning Mediatech, Inc), 1% sodium pyruvate (Corning Mediatech, Inc), 1% L-glutamine (Corning Mediatech, Inc), and 2.5 μg/ml plasmocin (InvivoGen, Inc). All cells were grown in a humidified atmosphere in a 5% CO_2_ incubator.

### Transduction constructs

Transduction constructs were produced as previously described (12, 60, 63). Sections of the coding region for PBRM1-containing BD4 mutations were synthesized (Biomatik) and inserted into the digested TetO-Fuw-PBRM1 WT plasmid (Addgene plasmid #85746) (63) using the In-FusionHD cloning kit (Clontech Laboratories, Inc) and confirmed by WideSeq (Purdue University). A TetO-Fuw empty vector (Addgene plasmid #85747) (63) was also used. The dual reporter construct pFU-Luc2-eGFP (L2G) (77) was a gift from Huiping Liu. Lentiviral particles for pLenti CMV rtTA3 Hygro (w785-1) (Addgene plasmid #26730) were a gift from Eric Campeau.

### Lentiviral infection

HEK293T cells were transfected with lentivirus constructs along with packaging vectors pMD2.G and psPAX2. After 48 h, the supernatant was collected and concentrated by ultracentrifugation (17,000 rpm for 2 h) and resuspended in 200 μl of PBS. Caki-2 cells were infected with concentrated virus using spinfection (where cells were centrifuged at 1500 rpm in a swinging bucket rotor for 1 h). Fresh medium was added 16 h after infection, and cells were allowed to recover for 24 h before selection. Caki-2 cells were selected for 2 weeks with puromycin (2 μg/ml) (Sigma-Aldrich) and hygromycin (200 μg/ml) (Corning Mediatech) to ensure stable transduction.

### GFP-Caki-2 and PBRM1^+^-Caki-2 cell line generation

Caki-2 cells expressing GFP and Caki-2 cells re-expressing PBRM1 were generated as previously described (63). GFP expression was performed by transducing Caki-2 parental cells with lentiviral particles for the dual reporter construct pFU-Luc2-eGFP (L2G) (77). GFP-expressing cells were selected using fluorescence-activated cell sorting. GFP^+^ Caki-2 cells were then transduced with lentiviral particles for pLenti CMV rtTA3 Hygro (w785-1) (Addgene plasmid #26730) for tetracycline-inducible expression and selected as described above. Upon selection, cells were transduced with lentiviral particles for TetO-Fuw empty vector (Addgene plasmid #85747). PBRM1 reexpression was performed by transducing Caki-2 parental cells with lentiviral particles for pLenti CMV rtTA3 Hygro (w785-1) (Addgene plasmid #26730) for tetracycline-inducible expression and selected as described above. Upon selection, cells were transduced with lentiviral particles for TetO-Fuw empty vector (Addgene plasmid #85747), TetO-Fuw-PBRM1 WT (Addgene plasmid #85746), or TetO-Fuw-PBRM1-BD4 missense variants. All Caki-2 cells were cultured with 2 μg/ml doxycycline (EMD Chemicals) for at least 72 h before and throughout the experiments to induce protein expression. Cell lines were free of *mycoplasma* contamination for all experiments.

### Immunoprecipitation

Immunoprecipitation was performed as previously described (64). Caki-2 cells (1 × 10^7^) were harvested and lysed in 2 ml of buffer A (20 mM Hepes pH 7.9, 25 mM KCl, 0.1% v/v Nonidet P-40, 10% v/v glycerol) plus 1:1000 leupeptin, pepstatin A, and aprotinin protease inhibitors (Cayman Chemical) and centrifuged at 600*g* for 10 min. The nuclei were then resuspended in 250 μl of immunoprecipitation (IP) buffer (25 mM Tris pH 8.0, 300 mM NaCl, 1% v/v Nonidet P-40, 1 mM EDTA, plus protease inhibitors) and rotated at 4 °C for 30 min. The extracts were cleared by centrifugation at 21,000*g* for 30 min. The cleared extract was precleared with normal immunoglobulin G (IgG)-conjugated protein A/G magnetic beads (Pierce) for 20 min. One microgram of specific IgG was used per 0.2 mg lysate for immunoprecipitation. After overnight incubation, immunocomplexes were captured using protein A/G magnetic beads following a 2-h incubation. The beads were washed twice in chromatin IP buffer and three times in high stringency wash buffer (20 mM Hepes pH 7.9, 500 mM NaCl, 1% Triton X-100, 0.5% sodium deoxycholate, 1 mM EDTA). The proteins were eluted in 1× lithium dodecyl sulfate loading dye (Thermo Fisher Scientific) by heating at 70 °C for 10 min. Samples were heated at 95 °C for 10 min then loaded onto a 4 to 12% SDS-polyacrylamide gel (Invitrogen) for immunoblotting.

### Cycloheximide chase assay

Caki-2 cells (400,000 cells) were seeded in 6 cm dishes plates and under 2 μg/ml doxycycline for 3 days. Then cells were treated with 100 μg/ml cycloheximide (Selleck) for 0, 2, 6, 10, or 24 h. At each time point, cells were harvested and lysed in radioimmunoprecipitation assay buffer with 1:1000 leupeptin, pepstatin A, and aprotinin protease inhibitors (Cayman Chemical).

### Peptide pulldown

Peptide pulldowns were performed as previously described (12). Streptavidin Agarose Ultra Performance resin (15 μl) (Solulink) was washed three times with binding buffer (50 mM Tris pH 7.8, 150 mM NaCl, 0.5 mM DTT). The resin was resuspended in 300 μl of binding buffer with 2 μg of biotin-labeled peptide (AnaSpec) plus 1:1000 leupeptin, pepstatin A, and aprotinin protease inhibitors (Cayman Chemical), and samples were rotated at 4 °C for 1 h. The following peptides were used: H3(1–30), H3K14ac(1–30), and H3K14/18/23/27ac(1–30). Caki-2 cells (5 × 10^6^) were harvested and lysed in 2 ml of buffer A (20 mM Hepes pH 7.9, 25 mM KCl, 0.1% v/v Nonidet P-40, 10% v/v glycerol, plus protease inhibitors) and centrifuged. The nuclei were then resuspended in 250 μl of IP Buffer (25 mM Tris pH 8, 300 mM NaCl, 1% v/v Nonidet P-40, 1 mM EDTA, plus protease inhibitors) and rotated at 4 °C for 10 min. The samples were then spun down at 15,000 rpm for 15 min. The 250 μl of lysate was added to the peptide and resin solution and rotated overnight. The samples were washed for 10 min three times in binding buffer. The resin was resuspended in 1 × Bolt lithium dodecyl sulfate sample buffer (Invitrogen) and boiled for 5 min. Nuclear lysate input and the samples were loaded onto a 4 to 12% SDS-polyacrylamide gel (Invitrogen) for immunoblotting.

### Cell proliferation competition assay

Cell proliferation competition assays were performed as previously described (60). Doxycycline-induced GFP^+^/GFP^-^ Caki2 cells were seeded in a 1:1 ratio in 6-well plates. At 24 h postseeding, each well was trypsinized, and one-fourth of the harvested cells were reseeded in 6-well plates for the next time point, while three-fourths of the harvested cells were analyzed by flow cytometry to determine GFP^+^ and GFP^-^ populations. The 24-h GFP^-^/GFP^+^ ratio was used as a baseline for all the subsequent time points. The coculture wells were harvested every 72 to 96 h to maintain a confluency of <70%. Cell populations were analyzed using the Guava EasyCyte benchtop flow cytometer, with monoculture cells as the control. Data were analyzed with FlowJo (https://www.flowjo.com/) and GraphPad Prism.

### Quantitative real-time PCR

Quantitative real-time PCR was performed as previously described (12). Caki-2 cells were seeded at 50,000 cells/well in 6-well flat-bottom cell culture plates for 4 days under 2 μg/ml of doxycycline treatment. Cells were harvested and homogenized in TRIzol reagent (Thermo Fisher Scientific) for RNA extraction. Total RNA was reverse transcribed to complementary DNA with the Verso cDNA synthesis kit following the manufacturer’s instructions (Thermo Scientific). Quantitative real-time PCR was conducted using SYBR Green Mastermix (Thermo Fisher Scientific) on a Bio-Rad CFX connect real-time system. Primer sequences are in Table S5. The data were analyzed using the 2^(−ΔΔCT)^ method in GraphPad Prism. Each sample was tested in triplicate.

### Immunoblot

Immunoblotting was performed as previously described (12). Protein samples from cell lysates, coimmunoprecipitations, cycloheximide chase assays, and peptide pulldowns were quantified by BCA assays (78). After sample concentration was normalized, samples were denatured for 10 min at 95 °C, separated on a 4 to 12% SDS-polyacrylamide gel (Invitrogen), and transferred to a nitrocellulose membrane (Millipore). The membrane was blocked with 5% w/v bovine serum albumin (VWR International) in PBS containing 0.1% v/v Tween 20 for 1 h at room temperature and then incubated in a 1:1000 dilution of primary antibodies overnight at 4 °C. The primary antibodies were detected by incubating the membranes in a 1:10,000 dilution of goat-anti-rabbit or goat-anti-mouse IgG secondary antibodies (LI-COR Biotechnology) conjugated to IRDye 800CW (lot no. D20510-25) or IRDye 680 (lot no. D20920-25), respectively, for 1 h at room temperature. The signals were visualized using an Odyssey Clx imager (LI-COR Biotechnology). Any quantification was performed by band densitometry, normalizing to β-actin for the cycloheximide chase assays and to 10% input for the peptide pulldowns.

### Antibodies

Primary immunoblotting antibodies include β-Actin (Santa Cruz Biotechnology, sc-47778, lot no. J0421), BRG1 [EPNCIR111A] (Abcam, 110641, lot no. GR3375498-17), IgG (Cell Signaling, 2729S, lot no. 10), PBRM1 (Abcam, ab243876, lot no. GR3295469-1), TBP (Abcam, ab818, lot no. GR300917-1), and V5 (Invitrogen, 46-0705, lot no. 2378586).

## Data availability

All data are contained within the article or the Supporting information.

## Supporting information

This article contains supporting information.

## Conflict of interest

The authors declare they have no conflicts of interest with the contents of this article.
