# Supplementary material for: Cancer-associated polybromo-1 bromodomain 4 missense variants variably impact bromodomain ligand binding and cell growth suppression
Source: J Biol Chem. 2024 Mar 7;300(4):107146. doi: 10.1016/j.jbc.2024.107146 (PMC11002309; doi:10.1016/j.jbc.2024.107146)
Supplement: Supporting Information [file mmc1.pdf]

Supporting Information

Cancer-associated Polybromo-1 bromodomain 4 missense variants variably impact bromodomain ligand binding and cell growth suppression

Karina L. Bursch[1,2‡], Christopher J. Goetz[1‡], Guanming Jiao[3], Raymundo Nuñez[1], Michael D. Olp[1], Alisha Dhiman[3], Mallika Khurana[1], Michael T. Zimmermann[1,2,4], Raul A. Urrutia[1,2,5], Emily C. Dykhuizen[3], and Brian C. Smith[1,2,6]*

[1]Department of Biochemistry, Medical College of Wisconsin, Milwaukee, WI 53226, USA; [2]Structural Genomics Unit, Linda T. and John A. Mellowes Center for Genomic Sciences and Precision Medicine, Medical College of Wisconsin, Milwaukee, WI 53226, USA; [3]Department of Medicinal Chemistry and Molecular Pharmacology, Purdue University, West Lafayette, IN, 47907, USA; [4]Clinical and Translational Sciences Institute, Medical College of Wisconsin, Milwaukee, WI 53226, USA; [5]Department of Surgery, Medical College of Wisconsin, Milwaukee, WI 53226, USA; [6]Program in Chemical Biology, Medical College of Wisconsin, Milwaukee, WI 53226, USA

[‡]These authors contributed equally to this work

***Corresponding author:** Brian C. Smith, Email: brismith@mcw.edu

Supplementary Figures and Tables

Figure S1. Correlation between PBRM1-BD4 missense variant thermal shifts determined by SYPRO Orange thermal shift and Prometheus nanoDSF assays.

Figure S2. Circular dichroism spectra of PBRM1-BD4 missense variants.

Figure S3. PBRM1-BD4 wild type and PBRM1-BD4 missense variant normalized Alpha counts.

Figure S4. PBRM1-BD4 wild type and PBRM1-BD4 missense variant EMSA replicates.

Figure S5. Energy contributions to the *in silico* calculations of PBRM1-BD4 missense variant stability.

Figure S6. PBRM1-BD4 missense variants exhibit impaired cellular stability and acetylated histone peptide binding.

Figure S7. PBRM1-BD4 missense variants exhibit impaired cancer cell growth suppression.

Table S1. Classification of selected cancer-associated PBRM1-BD4 missense mutations.

Table S2. PBRM1-BD4 wild type and missense variant melting temperatures as determined by SYPRO Orange thermal shift assay.

Table S3. PBRM1-BD4 wild type and missense variant melting temperatures determined by Prometheus nanoDSF assay.

Table S4. PBRM1-BD4 missense variant binding ability to an H3K14ac peptide, as determined by AlphaScreen assay, normalized to PBRM1-BD4 wild type.

Table S5. RT-qPCR primer sequences.

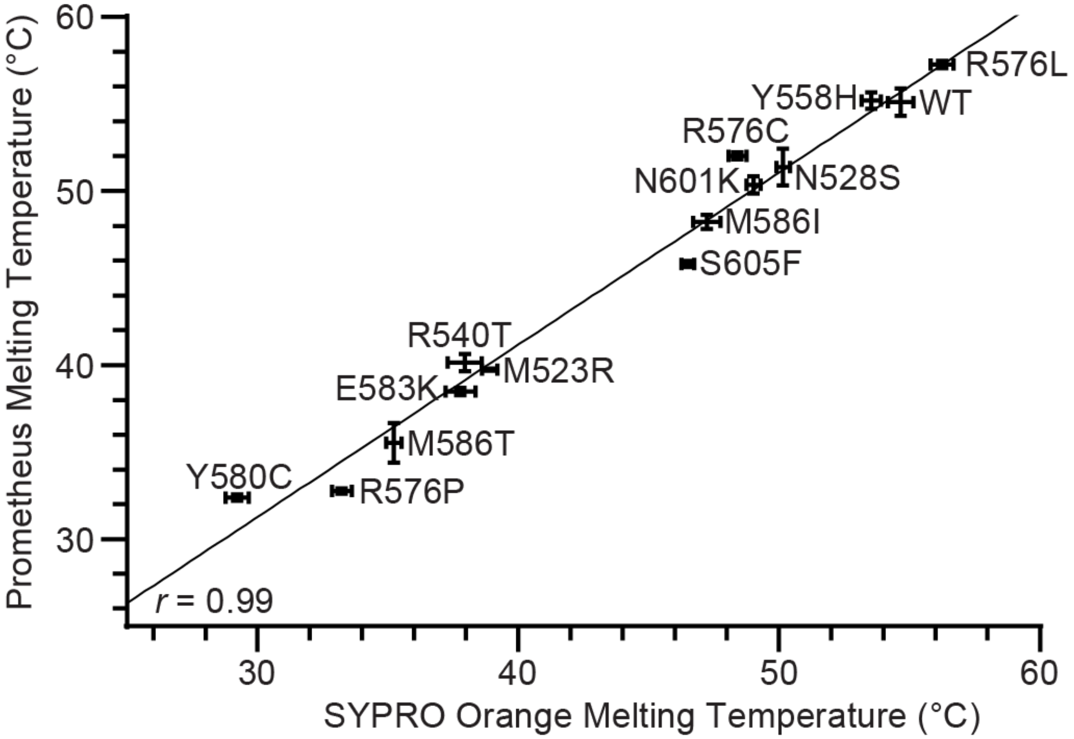

Figure S1. Correlation between PBRM1-BD4 missense variant thermal shifts determined by SYPRO Orange thermal shift and Prometheus nanoDSF assays, where horizontal error bars represent standard deviation of protein melting temperatures determined by the SYPRO Orange thermal shift assay and vertical error bars represent standard deviation of protein melting temperatures determined by the Prometheus nanoDSF assay.

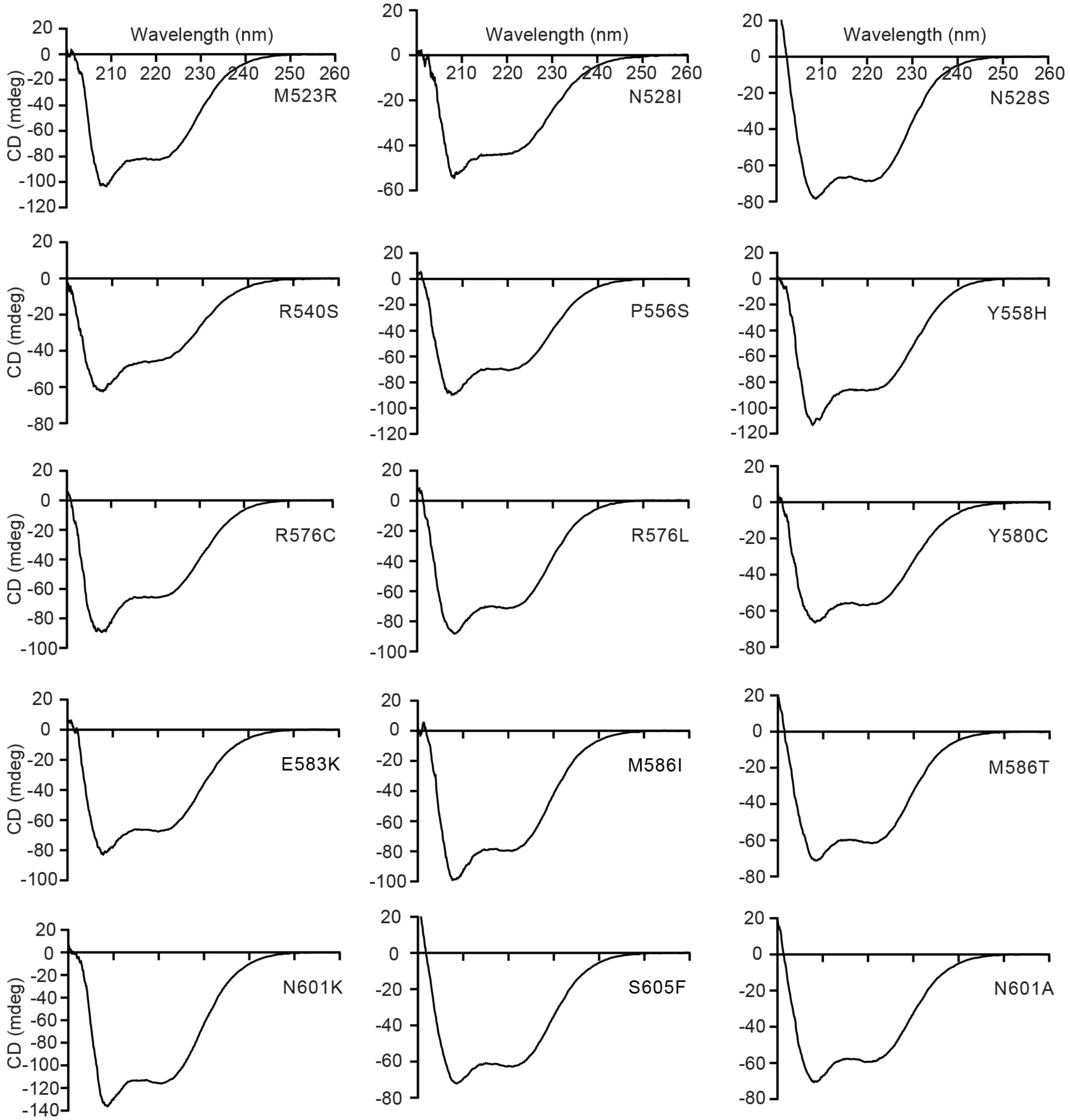

Figure S2. Circular dichroism spectra of PBRM1-BD4 missense variants.

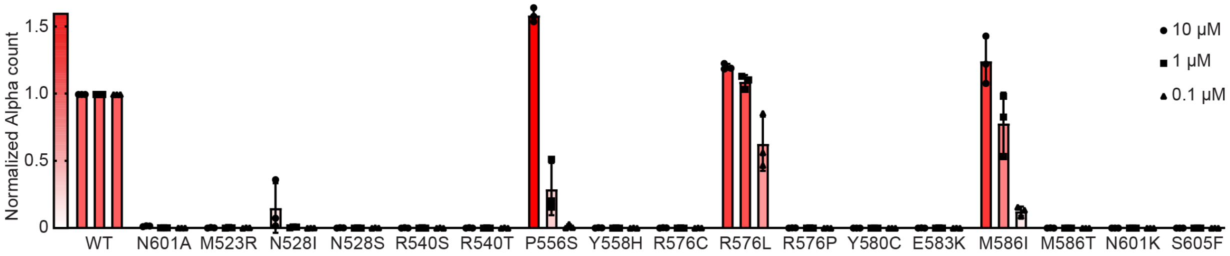

Figure S3. PBRM1-BD4 wild type and PBRM1-BD4 missense variant normalized Alpha counts.

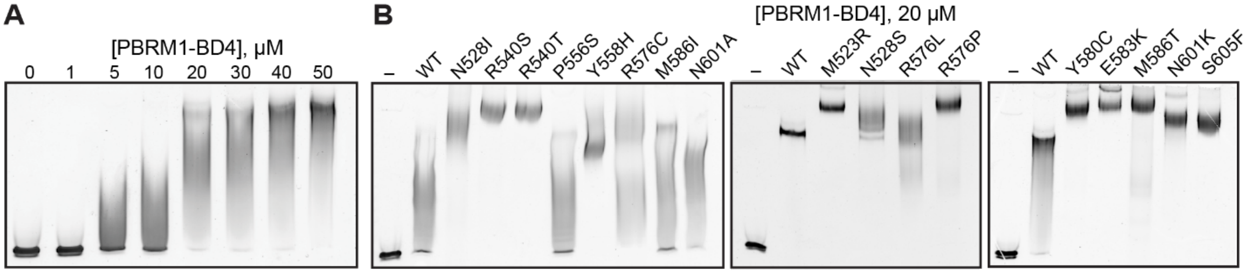

Figure S4. PBRM1-BD4 wild type and PBRM1-BD4 variant EMSA replicates. **(A)** EMSA replicate of PBRM1-BD4 wild type (0-50 μM) binding to 150 nM Widom 601 DNA. **(B)** EMSA replicate of PBRM1-BD4 variants (20 μM) binding to 150 nM Widom 601 DNA.

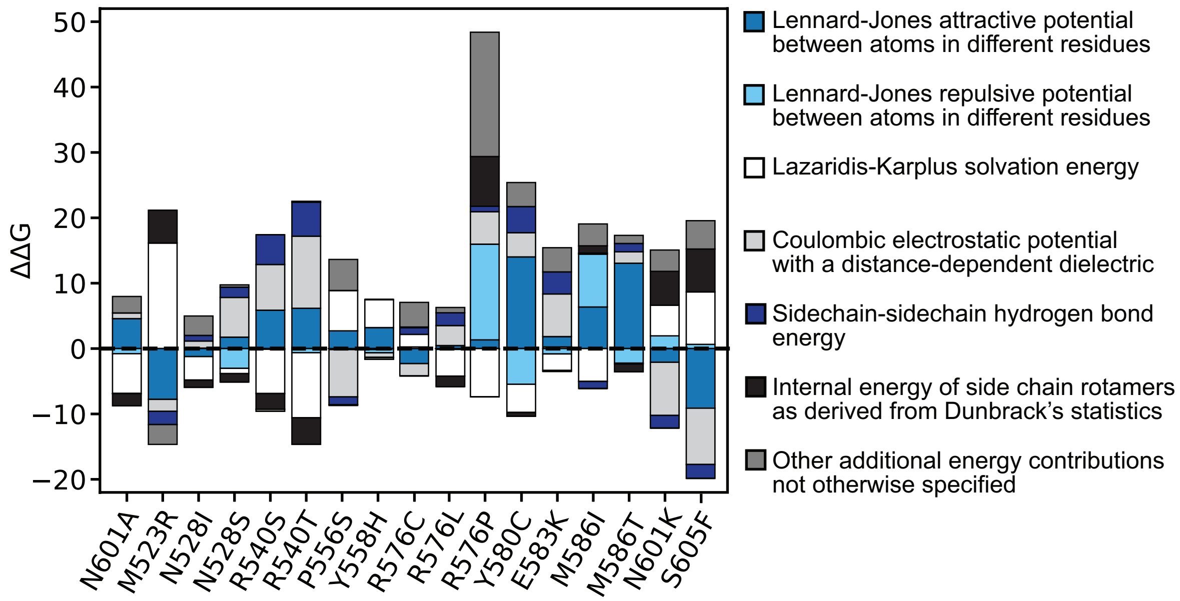

Figure S5. Energy contributions to the *in silico* calculations of PBRM1-BD4 missense variant stability. Lennard-Jones attractive potential between atoms in different residues (azure); Lennard-Jones repulsive potential between atoms in different residues (light blue); Lazaridis-Karplus solvation energy (white); Coulombic electrostatic potential with a distance-dependent dielectric (light gray); sidechain-sidechain hydrogen bond energy (dark blue); internal energy of side chain rotamers as derived from Dunbrack's statistics (black); other additional energy contributions not otherwise specified (dark gray).

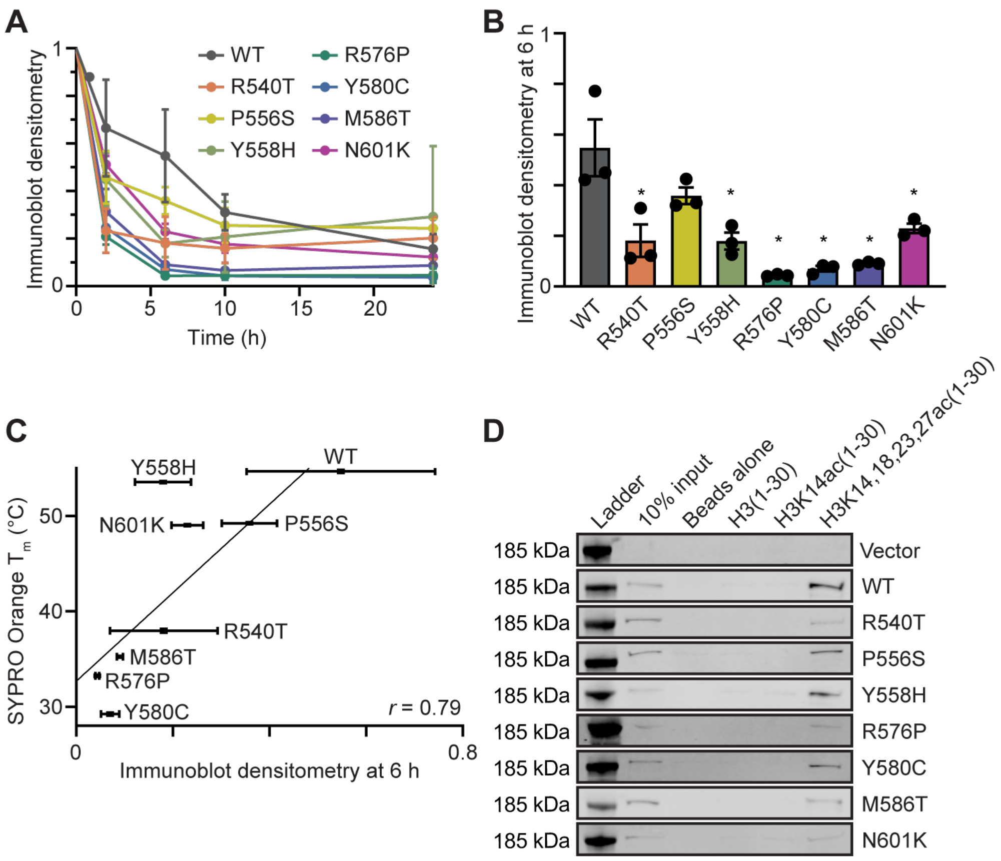

Figure S6. PBRM1-BD4 missense variants exhibit impaired cellular stability and acetylated histone peptide binding. **(A)** Immunoblot densitometry quantitation of full cycloheximide chase assay time course for PBRM1-BD4 wild type and PBRM1-BD4 missense variants, where error bars represent the standard deviation. **(B)** Immunoblot densitometry quantitation of PBRM1-BD4 wild type and PBRM1-BD4 missense variants at 6 hours of cycloheximide exposure, where error bars represent the standard error of the mean. **(C)** Correlation of PBRM1-BD4 wild type and PBRM1-BD4 missense variant protein stability as assessed by the cellular cycloheximide chase assay at 6 hours and the biophysical SYPRO Orange thermal shift assay, where horizontal error bars represent standard deviation of immunoblot densitometry quantitation at 2 hours of cycloheximide exposure and vertical error bars represent standard deviation of protein melting temperatures determined by the SYPRO Orange thermal shift assay. **(D)** Replicate of acetylated histone H3 peptide pulldown by PBRM1 wild type and PBRM1-BD4 missense variants.

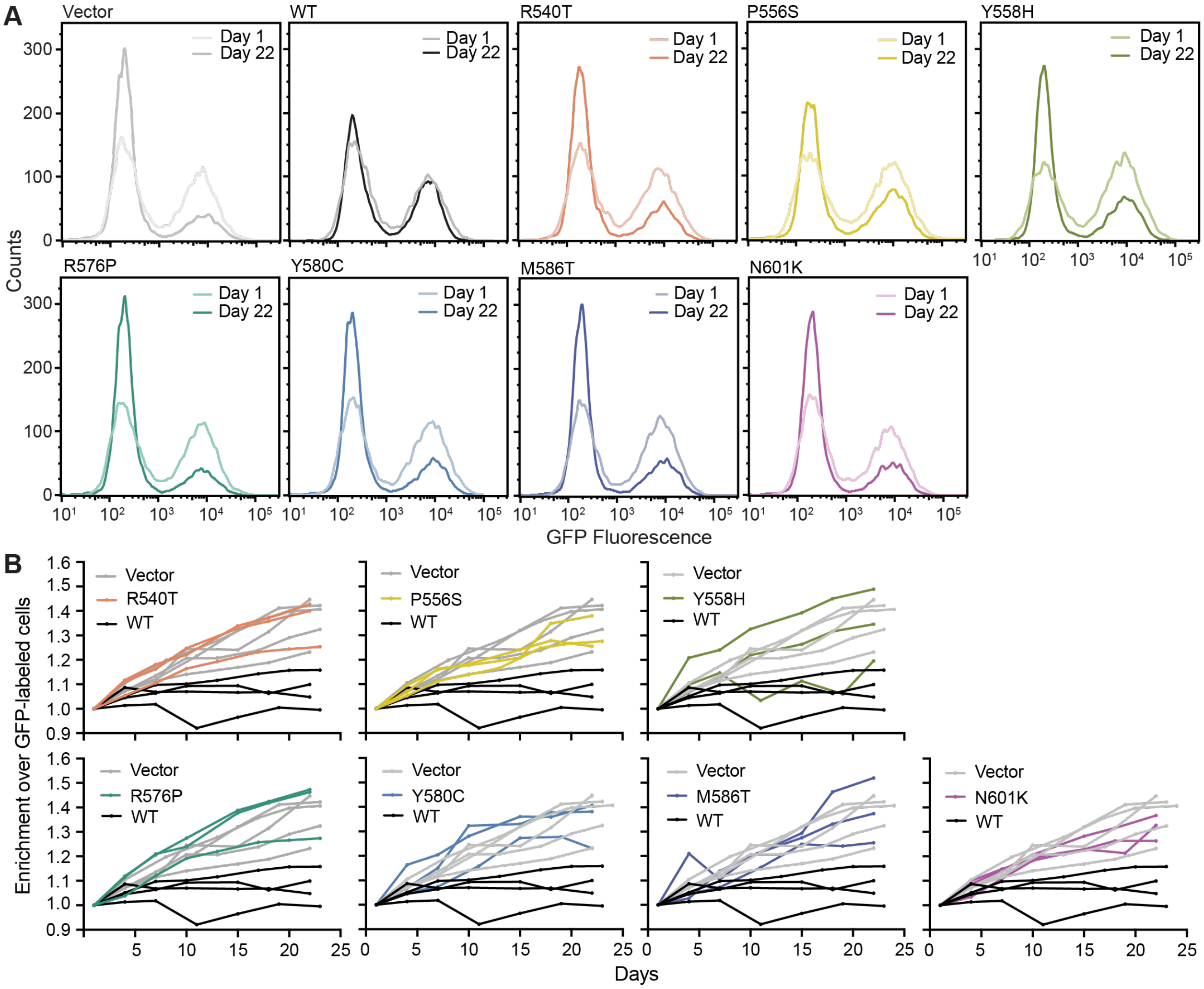

Figure S7. PBRM1-BD4 missense variants exhibit impaired cancer cell growth suppression. **(A)** Flow cytometry histograms of Caki-2 PBRM1 WT and PBRM1-BD4 missense variant cell abundance compared to GFP-labeled Caki-2 cells lacking PBRM1 at Day 1 versus Day 22. **(B)** Growth curves of Caki-2 PBRM1 WT and PBRM1-BD4 missense variant cell growth normalized to the growth of GFP-labeled Caki-2 cells lacking PBRM1 over 22 days (n=5 for vector, n=4 for WT, n=3 for missense variants).

Table S1. Classification of selected cancer-associated PBRM1-BD4 missense mutations.

Missense mutation	Classification
M523R	residue mutated in ≥3 patients
N528I	residue mutated in ≥3 patients
N528S	
R540S	conserved residue
R540T	
P556S	conserved residue
Y558H	conserved residue
R576C	residue mutated in ≥3 patients
R576L	
R576P	
Y580C	conserved residue
E583K	residue mutated in ≥3 patients
M586I	residue mutated in ≥3 patients
M586T	
N601K	conserved residue
S605F	conserved residue and mutated in ≥3 patients

Table S2. PBRM1-BD4 wild type and missense variant melting temperatures as determined by SYPRO Orange thermal shift assay.

PBRM1-BD4	Mean T_m	SD of mean T_m	Mean ΔT_m	SD of Mean ΔT_m	n
WT	54.7	0.5	0.0	0.0	9
N601A	57.3	0.2	2.6	0.4	9
M523R	38.9	0.3	-15.7	0.7	9
N528I	41.7	0.3	-12.9	0.7	9
N528S	50.2	0.3	-4.5	0.6	9
R540S	41.2	0.7	-13.5	1.0	9
R540T	38.0	0.6	-16.7	0.8	9
P556S	49.2	0.3	-5.4	0.8	9
Y558H	53.5	0.4	-1.1	0.8	9
R576C	48.4	0.3	-6.3	0.7	9
R576L	56.2	0.4	1.6	0.7	9
R576P	33.2	0.4	-21.4	0.5	9
Y580C	29.2	0.4	-25.4	0.4	9
E583K	37.8	0.6	-16.9	0.8	9
M586I	47.2	0.5	-7.4	0.7	9
M586T	35.2	0.3	-19.4	0.4	9
N601K	49.0	0.3	-5.9	0.2	6
S605F	46.5	0.2	-8.2	0.5	9

Table S3. PBRM1-BD4 wild type and missense variant melting temperatures determined by Prometheus nanoDSF assay.

**PBRM1-BD4**	**Mean T_m**	**SD of mean T_m**	**n**
WT	55.1	0.8	6
M523R	39.7	0.1	3
N528S	51.4	1.1	3
R540T	40.2	0.5	3
Y558H	55.2	0.5	3
R576L	57.3	0.2	3
R576P	32.8	0.1	3
R576C	52.0	0.2	3
Y580C	32.4	0.2	3
E583K	38.5	0.2	3
M586I	48.2	0.4	3
M586T	35.5	1.1	3
N601K	50.4	0.5	3
S605F	45.8	0.2	3

Table S4. PBRM1-BD4 missense variant binding ability to an H3K14ac peptide, as determined by AlphaScreen assay, normalized to PBRM1-BD4 wild type.

	0.1 µM PBRM1-BD4		1 µM PBRM1-BD4		10 µM PBRM1-BD4		
PBRM1-BD4	Mean Alpha count	SD of mean Alpha count	Mean Alpha count	SD of mean Alpha count	Mean Alpha count	SD of mean Alpha count	n
WT	1.00	—	1.00	—	1.00	—	3
N601A	0.01	—		—		—	3
M523R	0	—	—	—	0	—	3
N528I	0	—	0.01	—	0.15	0.18	3
N528S	0	—	0	—	0	—	3
R540S	0	—	0	—	0	—	3
R540T	0	—	0	—	0	—	3
P556S	0.01	0.02	0.29	0.20	1.59	0.05	3
Y558H	0	—	0	—	0	—	3
R576C	0	—	0	—	0	—	3
R576L	0.63	0.20	1.09	0.05	1.21	0.02	3
R576P	0	—	0	—	0	—	3
Y580C	0	—	0	—	0	—	3
E583K	0	—	0	—	0	—	3
M586I	0.13	0.03	0.78	0.23	1.25	0.18	3
M586T	0	—	0	—	0	—	3
N601K	0	—	0	—	0	—	3
S605F	0	—	0	—	0	—	3

— indicates a mean Alpha count or standard deviation of 0.

Table S5. RT-qPCR primer sequences.

Gene Name	Forward Primer	Reverse Primer
UBC	ATTTGGGTCGCGGTTCTTG	TGCCTTGACATTCTCGATGGT
HACE1	AGTTGCCCGAGGATAATGAAAC	TCCACCGATCCACAATTTGCT
GLRX	CCCATCAAACAAGGGCTTCTG	CTGCATCCGCCTATACAATCTT
MOXD1	TCTACAGACCAGTCACGACCT	CCTTGAATCGACCACTCAGCA
THBS1	AGACTCCGCATCGCAAAGG	TCACCACGTTGTTGTCAAGGG
FBN1	TTTAGCGTCCTACACGAGCC	CCATCCAGGGCAACAGTAAGC
YWHAZ	TGCTTGCATCCCACAGACTA	AGGCAGACAATGACAGACCA
